# Comparison and aggregation of event sequences across ten cohorts to describe the consensus biomarker evolution in Alzheimer’s disease

**DOI:** 10.1186/s13195-022-01001-y

**Published:** 2022-04-20

**Authors:** Sepehr Golriz Khatami, Yasamin Salimi, Martin Hofmann-Apitius, Neil P. Oxtoby, Colin Birkenbihl, for the Alzheimer’s Disease Neuroimaging Initiative, the Japanese Alzheimer’s Disease Neuroimaging Initiative, the Alzheimer’s Disease Repository Without Borders Investigators

**Affiliations:** 1 Department of Bioinformatics, Fraunhofer Institute for Algorithms and Scientific Computing (SCAI), 53757 Sankt Augustin, Germany; 2 Bonn-Aachen International Center for Information Technology (B-IT), University of Bonn, 53115 Bonn, Germany; 3 Centre for Medical Image Computing and Department of Computer Science, University College London, Gower St, London, WC1E 6BT UK

**Keywords:** Alzheimer’s disease, Event-based models, Biomarker ordering, Disease progression, External validation, Meta-sequence

## Abstract

**Background:**

Previous models of Alzheimer’s disease (AD) progression were primarily hypothetical or based on data originating from single cohort studies. However, cohort datasets are subject to specific inclusion and exclusion criteria that influence the signals observed in their collected data. Furthermore, each study measures only a subset of AD-relevant variables. To gain a comprehensive understanding of AD progression, the heterogeneity and robustness of estimated progression patterns must be understood, and complementary information contained in cohort datasets be leveraged.

**Methods:**

We compared ten event-based models that we fit to ten independent AD cohort datasets. Additionally, we designed and applied a novel rank aggregation algorithm that combines partially overlapping, individual event sequences into a meta-sequence containing the complementary information from each cohort.

**Results:**

We observed overall consistency across the ten event-based model sequences (average pairwise Kendall’s tau correlation coefficient of 0.69 ± 0.28), despite variance in the positioning of mainly imaging variables. The changes described in the aggregated meta-sequence are broadly consistent with the current understanding of AD progression, starting with cerebrospinal fluid amyloid beta, followed by tauopathy, memory impairment, FDG-PET, and ultimately brain deterioration and impairment of visual memory.

**Conclusion:**

Overall, the event-based models demonstrated similar and robust disease cascades across independent AD cohorts. Aggregation of data-driven results can combine complementary strengths and information of patient-level datasets. Accordingly, the derived meta-sequence draws a more complete picture of AD pathology compared to models relying on single cohorts.

**Supplementary Information:**

The online version contains supplementary material available at 10.1186/s13195-022-01001-y.

## Background

Alzheimer’s disease, in combination with its clinical manifestation/syndrome (AD) [1], is a progressive, multifaceted disease whose cognitive symptoms surface years after disease onset [2]. In order to identify crucial opportunities for medical interventions that could potentially prevent or delay symptoms, it is vital to understand the temporal relationship of pathological changes underlying the progressive nature of AD. To this end, cognitive assessments and a wide range of biomarkers, including cerebrospinal fluid (CSF) markers and neuroimaging-derived measures, have been established to monitor the disease’s progression. Measuring these markers enables the observation of biochemical, structural, functional, and cognitive changes that occur as the disease progresses [3] and the resulting data can build the basis for data-driven approaches that aim to determine the relative temporal dependencies between biomarkers and cognitive symptoms [4]. Previously, a variety of data-driven models have been developed with the aim of accomplishing this task [5–10].

One model archetype that has found wide success in the context of neurodegenerative diseases [11–14] and AD specifically [15] is the event-based model (EBM) [13]. It is a data-driven probabilistic generative model that characterizes the progression of a disease in the form of a single sequence of events which describes the relative order of measured markers turning from a normal state to a diseased state (i.e., abnormal state). Such event sequences carry the benefit that they are highly interpretable and, although describing disease progression, can already be learned from cross-sectional cohort study data. Previously, EBMs have been used to derive event sequences [13], stage subjects in their disease progression [15], predict conversion from one clinical stage to the other (i.e., cognitively unimpaired (CU) to mild cognitive impairment (MCI), or MCI to AD) [16], and uncover disease phenotypes with distinct temporal progression patterns.

To build an EBM, patient-level data are needed on which the model can be fit. In recent decades, an increasing number of observational cohort studies have released their collected data for research purposes, including the Alzheimer’s Disease Neuroimaging Initiative (ADNI) [17], the European Prevention of Alzheimer’s Dementia (EPAD) [18], and AddNeuroMed [4]. So far, however, only a few studies in the AD domain have applied EBMs to data from other cohorts besides ADNI [19, 20]. Previous work evaluating data-driven progression modeling based on cohort datasets has shown that the participant recruitment procedures can introduce cohort-specific systematic statistical biases into the collected data [21], which, in turn, can bias the estimation of disease progression [22]. Therefore, it is necessary to replicate and validate data-driven results in independent cohorts to ensure robust conclusions. Consequently, it remains unclear whether event sequences determined from one cohort dataset would generalize beyond the discovery cohort itself and, further, if sequences generated across several cohorts were concordant among each other. Simultaneously, gaining a comprehensive event sequence combining all relevant AD biomarkers, cognitive assessments, and functional scores is infeasible, since cohort studies can only measure a limited set of variables that are often only partially overlapping between them [23]. In theory, however, this allows for an estimation of individual event sequences from distinct cohorts which cover complementary sets of markers. Aggregating results across cohorts would harness this complementary information by assembling a meta-sequence that provides a more complete picture of the development and progression of AD.

In this work, we present a systematic, in-depth comparison of AD event sequences derived from ten independent landmark cohort studies to investigate the generalizability and robustness of EBM-derived AD progression patterns. Furthermore, we designed a novel rank aggregation algorithm which we used to aggregate the event sequences into a single meta-sequence, thereby fusing the complementary information in all variables assessed across the studies. Our work harnesses the heterogeneity in cohort study designs and measurements to produce a meta-sequence providing a more complete, and robust, picture of the temporal order of pathological marker changes in AD progression.

## Methods

### Investigated cohort datasets

We selected ten independent AD cohort studies for our analysis by systematically exploring suitable datasets using the ADataViewer [23]. The prerequisite for including a cohort into our analysis was that (1) diagnostic staging into CU, MCI, and AD was performed [24]; (2) cross-sectional data was available for at least 10 patients per diagnostic group; and (3) multiple data modalities were collected. The cohorts that were ultimately selected are presented in Table 1. All cohorts followed the NINCDS-ADRDA diagnostic criteria [24].

**Table 1:** Selected cohorts, their number of participants per disease stage, and their number of considered variables

Cohort	Consortium	CU	MCI	AD	Total	Number of CSF, PET, and imaging biomarkers	Number of cognitive tests
ADNI [17]	The Alzheimer’s Disease Neuroimaging Initiative	38	63	35	136	9	9
JADNI [25]	Japanese Alzheimer’s Disease Neuroimaging Initiative	17	87	10	114	9	9
AIBL [26]	The Australian Imaging, Biomarker Lifestyle Flagship Study of Ageing	92	23	13	128	0	10
NACC [27]	The National Alzheimer’s Coordinating Center	24	42	24	90	9	7
ANM [28]	AddNeuroMed	120	161	103	384	6	1
EMIF-1000 [29]	European Medical Information Framework	47	229	53	329	4	5
EDSD [30]	European DTI Study on Dementia	26	34	32	92	5	7
ARWIBO [31]	Alzheimer’s Disease Repository Without Borders	214	115	38	367	7	3
OASIS-1 [32], OASIS-2 [33]	Open Access Series of Imaging Studies	135	70	30	235	6	1
WMHAD [34]	White Matter Hyperintensities in Alzheimer’s Disease	19	27	42	88	6	7

### Variable selection

We aimed at including a wide spectrum of variables to uncover the temporal relationship across multimodal markers of AD pathology that capture, for example, different biochemical, cognitive, or structural changes. In order to include a specific variable, it must have been measured in at least the CU and AD groups of the respective study to allow for later modeling. Furthermore, only a minimal amount of missing values was tolerable, as participants with missing values in any of the ultimately selected variables had to be excluded from the analysis. This led to a trade-off between the inclusion of an increasing number of variables and the amount of participants available for analysis. We present an example of variable inclusion and the effect on sample size in the supplementary material (Table S1). In total, 36 unique variables were selected from different data modalities covering neuropsychological and cognitive tests, CSF markers, and MRI-derived brain region volumes. The complete list of selected biomarkers and their corresponding modality are presented in Table 2. The number of variables per cohort is given in Table 1.

**Table 2:** The selected biomarkers and their corresponding abbreviations

Modality	Biomarker	Abbreviation	Number of cohorts containing variable
**Clinical assessments**	Neuropsychiatric Inventory	NPI	2
	Logical Memory - Delayed Recall Total Number of Story Units Recalled	LDEL	5
	Alzheimer’s Disease Assessment Scale (13-items)	ADAS13	2
	Alzheimer’s Disease Assessment Scale (11-items)	ADAS11	2
	Logical Memory - Immediate Recall Total Number of Story Units Recalled	LIMM	6
	Trail Making Test-B	TRABS	2
	Digit-Symbol Coding Test	DIGITS	2
	California Verbal Learning Test Delayed Raw Score	LIDE	1
	Category Fluency (animals - fruits/vegetables)	CATFLU	3
	Figure Copy	FIGC	3
	California Verbal Learning Test Recall Raw Score	LIRE	2
	Figure recall	FIGR	2
	C/D Stroop Test Raw	STROOP	1
	Short Term Memory	STM	1
	Language	LANGU	1
	Perceptual Orientation	ORIENT	2
	Mental Manipulation	MENMA	1
	Attention	ATTEN	1
	Clock Drawing Test Total Score	CLKS	2
	Executive Memory	EXECUTIVE	1
	Word List Learning Trial	LICOR	1
	Boston Naming Test Score	BNTS	2
	Digit Symbol Substitution Test	WAIS	2
**CSF markers**	Amyloid-β	ABETA	4
	Total tau	TAU	4
	Phosphorylated tau (p-Tau)	PTAU	4
**Imaging markers**	Entorhinal volume	ENTOR	8
	Hippocampal volume	HIPPO	8
	Fusiform volume	FUSIF	8
	Ventricles volume	VENT	8
	Middle temporal volume	MIDTEPM	8
	Accumulated CSF in the brain	CSFVOL	5
	Fluorodeoxyglucose positron emission tomography (FDG PET)	FDG	2

### Participants

An available diagnosis of a participant as either CU, MCI, or AD was a prerequisite for inclusion. Furthermore, any participant with a diagnosis of cognitive impairment that was not linked to AD by the respective study’s clinicians was excluded. Furthermore, only participants with complete data across all selected biomarkers could be used in our modeling approach. The number of participants per cohort and diagnostic group is described in Table 1.

### Progression modeling via event-based models

The EBM derives a probabilistic sequence from patient-level data that describes the temporal order in which measured values of variables turn from a normal to an abnormal state. Each of these transitions is called an event. In this context, normality or abnormality are defined non-parametrically using kernel density estimation mixture modeling on the empirical values of the modeled cohort’s CU and AD populations, respectively [35]. This probabilistic allocation of measurements into two groups allows study participants (in particular, patients) to have a mix of occurred and non-occurred events across all measurements which lays the foundation to estimate the most likely event sequence. Here, the EBM assumes that the biomarkers monotonically change towards abnormality as the disease progresses and that this process is irreversible. Furthermore, there are no a priori assumptions regarding predefined disease stages, cut points determining the abnormality of biomarkers, or the temporal relationship between them. The most likely sequence of events *S* is then estimated by maximizing the likelihood (𝑋|𝑆) (Eq. 1), where variable measurements are denoted by *x* ∈ *X* for *i* ∈ *M* markers and *j* ∈ *N* indicates the individual samples.

1 $$\mathit{\Pr}\left(X|S\right)=\prod \limits_{j=1}^N\left[\sum \limits_{m=0}^M\left\{\prod \limits_{i=1}^m\mathit{\Pr}\left({x}_{ij}|{E}_i\right)\prod \limits_{i=m+1}^M\mathit{\Pr}\Big({x}_{ij}|\neg {E}_i\Big)\right\}\right]$$

Here, *Pr*(*x*_*ij*_| *E*_*i*_) and *Pr*(*x*_*ij*_ ∣  ¬ *E*_*i*_) describe the probability of observing the value of *x* given that the event *E*_*i*_ (i.e., variable *x* turning abnormal) has, or has not, occurred, respectively. For more details, we refer to the Supplementary Material and the original publication of the KDE EBM by Firth et al. [35]. The derived mixture models per cohort and measurement are presented in Fig. S3.

To quantify the similarity of distinct event sequences, we calculated the pairwise Kendall’s tau rank correlation coefficient (KTC) across sequences and the Bhattacharrya coefficient (*BC*) for specific events as explained in Oxtoby et al. [12]. The KTCs were calculated pairwise across all cohorts while considering only the relative ranks of variables which were common among the respective two cohorts’ sequences. An average KTC that is close to 1 and shows low standard deviation across the cohorts would indicate high concordance. An average BC close to 1 implies high similarity in the positional variance of ranks while the BC amounts to 0 for completely different patterns.

### Generating a meta-sequence based on event sequences derived from multiple cohort studies

To generate a meta-sequence, we propose a method that combines individual event sequences (called “base sequences”) stemming from independent datasets. We assemble a meta-sequence in a two-step procedure: first, building on the ideas presented in [36] and [37], we generate all possible sequences comprising *k* variables that are randomly drawn from the union of variables encountered in the base sequences (with *k* < total number of variables). The generated sequence with the minimum average distance to all base sequences is selected as a starting sequence for the next step. In step 2, this starting sequence is extended by iteratively adding the remaining variables to it (i.e., those not in the *k* variables of the starting sequence), such that the average distance between the altered sequence and all base sequences remains minimal. Here, the new variable is not necessarily added to the end of the sequence but all possible positions are considered. This process is repeated until all variables have been included into the sequence which finally forms the aggregated meta-sequence. Therefore, the algorithm is deterministic once the base sequences are calculated. Splitting the algorithm in two steps (an exhaustive search for the first *k* variables followed by the greedy insertions) was necessary, as the search space (i.e., all possible meta-sequences) grows exponentially with the number of variables in the base sequences. Further explanations about the algorithm, the handling of partially overlapping lists, and access to the corresponding python code are provided in the Supplementary Material and Fig. S1.

We designed and applied two algorithms for generating a meta-sequence: one based on the maximum likelihood (ML) sequences presented by EBMs and one relying on bootstrapping. In the former, only the ML base sequences of each cohort were used as an input to our algorithm. Therefore, however, solely the rank of each event is considered while its positional variance within a sequence is not taken into account.

During the bootstrapping approach, all base sequences are resampled *b*-times with replacement. This means that a new base sequence is generated per cohort based on a sample of that cohort’s participants that was randomly drawn with replacement and is of equal size to the original cohort. For each of these *b* sets of base sequences, one meta-sequence is generated. The resulting consensus over the *b* meta-sequences is visualized using a positional variance diagram which displays the variation in event ranks exhibited across the generated meta-sequences.

For this work, we generated a meta-sequence considering only variables which were present in at least three cohorts (Table 2) and set *k* equal to eight. In our bootstrapped version, we drew 500 bootstrap samples. The distance metric chosen was Spearman’s footrule distance which takes the absolute difference in positions of variables into account.

### Patient staging according to the determined meta-sequence

Once a meta-sequence was determined, one possible way to evaluate its plausibility across cohorts was to evaluate the assignment of subjects of the respective cohorts to the disease stages defined by the meta-sequence. In this process, each participant of a study was assigned to a disease stage which represents the current step in the meta-sequence at which the participant most likely resides. Therefore, stage 0 refers to the absence of any abnormal markers, while the farthest progressed stage *m* (with *m* being equal to the length of the sequence) implies that all events occurred for that particular subject. The corresponding equation underlying the patient staging is provided in the Supplemental Material.

Here, we staged only participants from cohorts that contained measurements of all investigated modalities (i.e., ADNI, JADNI, EMIF, and NACC) and were bound to consider only those variables of the meta-sequence that were found in the respectively staged cohort.

## Results

### Comparing event sequences derived from multiple cohort studies

**Fig. 1:**
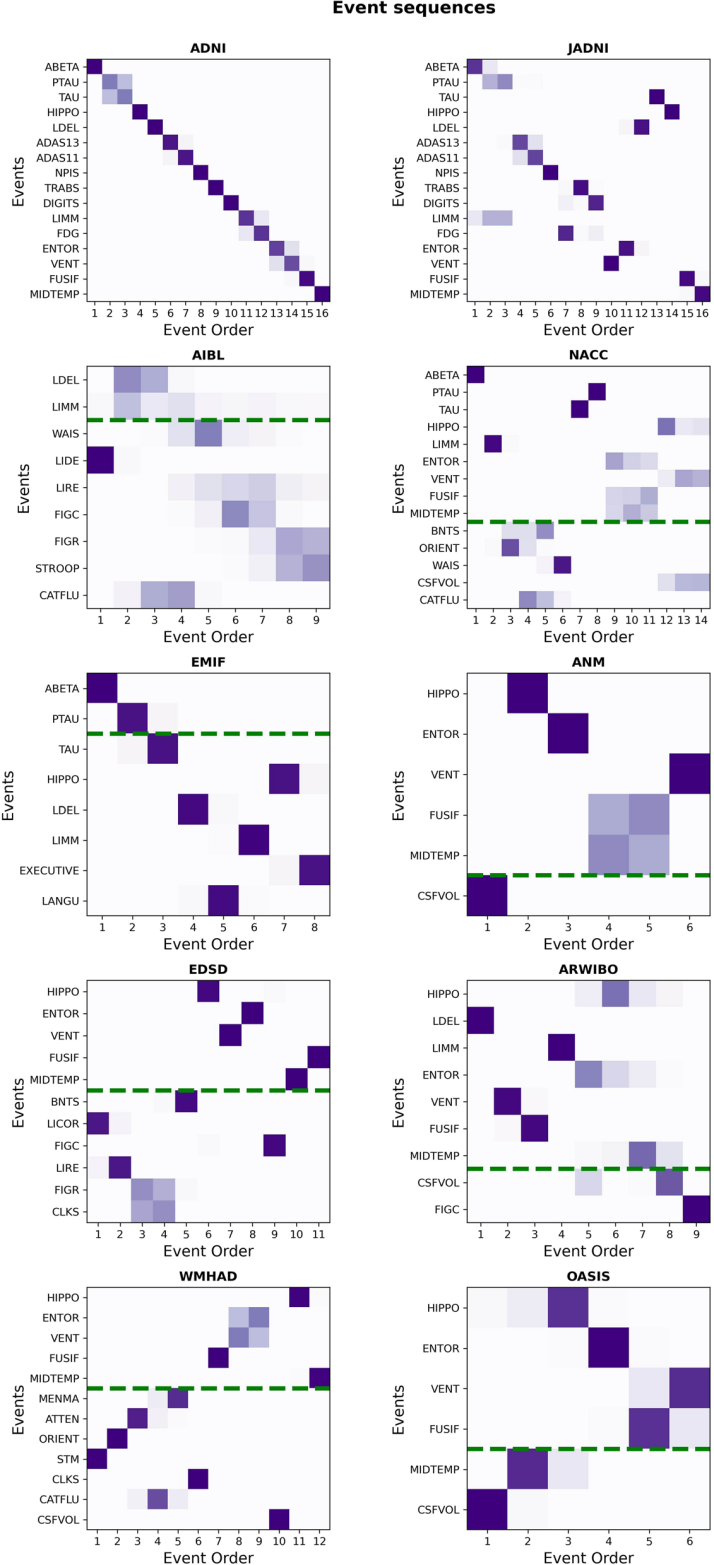
Individual event sequences estimated from the ten investigated cohorts. To facilitate the comparison of relative event positions, the *y*-axes follow the ADNI sequence. Common events between ADNI and the other cohorts are presented above a dashed green line. The closer the sequences are to the ADNI sequence, the more diagonal the probabilistic position (colored squares) will align from top-left to bottom-right. Lateral shifts due to additional events which were not available in ADNI have to be disregarded (as for example observed in WMHAD and EDSD). Event order 1 corresponds to the first position in the sequence. The shading of squares indicates the positional probability with darker shades corresponding to higher probabilities. The relative sizes of the squares do not encode any information. The event sequences in their original form are presented in Fig. S2

We observed broad consistency with respect to the position of events across all cohorts’ sequences which resulted in an average KTC of 0.69 ± 0.28 (pairwise KTCs are presented in Table S4; sequence similarity is also indicated visually through an approximately diagonal line of the event ranks from top-left to bottom-right in Fig. 1). In most cohorts’ sequences, CSF markers ranked highly, before cognitive impairments, which were again followed by MRI-derived brain volumes in the lower ranks.

The relative order among clinical assessments measuring different cognitive domains (e.g., memory, language, visuospatial, executive) was consistent across most cohorts (see Table S2 for a mapping of tests to cognitive domains). The cognitive impairment in all investigated cohorts started with memory dysfunction detected by logical memory tests (e.g., LDEL and LIMM), proceeded with language impairments exposed by tests such as the BNT and CATFLU. Thereafter, in most cohorts, visual dysfunction identified through the CLKS or FIGC followed, and finally, executive dysfunction recognized by, for example, the DIGIT and WAIS, occurred.

Among the cohorts where CSF biomarkers had been measured (ADNI, JADNI, EMIF, NACC), the relative positions of these biomarkers, in particular of tau (TAU) and phosphorylated tau (PTAU), varied. ABETA consistently placed first in all of these cohorts’ sequences, and TAU and PTAU were mainly found in early positions as well (ADNI, JADNI, and EMIF), with the exception of NACC where they placed in the middle of the sequence. However, in all cases except JADNI, PTAU and TAU were direct neighbors, indicating the consistent, direct link between them.

The relative order of the MRI-derived brain volume events was consistent across cohorts, albeit with some variance (average KTC of 0.64 ± 0.29 for MRI variables only). While the volume changes in ADNI, JADNI, ARWIBO, and WMHAD started with ventricular expansion and were then followed by atrophy of the temporal lobe (here, hippocampus, entorhinal, middle temporal, and fusiform gyrus), in other cohorts (ANM, OASIS, NACC, EDSD), atrophy of the temporal lobe regions was the first detected variables of the MRI modality. The position that was taken by each respective brain region varied again among the cohorts. However, in many cases, the probabilistic nature of the EBMs indicated that the order of MRI events could be interchangeable among themselves (average BC of 0.17 ± 0.13 for MRI variables only) and events occurred most probably in close temporal proximity or even simultaneously (Fig. S2), as far as the model could discern from the data.

The position of FDG-PET, another well-established imaging biomarker measuring brain hypometabolism, was consistent in both cohorts it was measured in (ADNI, JADNI). It preceded the MRI marker changes and occurred concurrently with clinical symptoms, being placed after logical memory tests such as the LIMM and LDEL. However, its positioning of FDG-PET related to assessments of executive function differed between the two cohorts.

### A multimodal meta-sequence of AD progression

To aggregate and investigate the complementary information from the base sequences in each cohort, we combined them into a single meta-sequence. Here, the position of a variable was determined based on its relative positions in all cohort sequences. Both versions of our algorithm (i.e., ML sequence-based and bootstrapping) were applied.

**Fig. 2:**
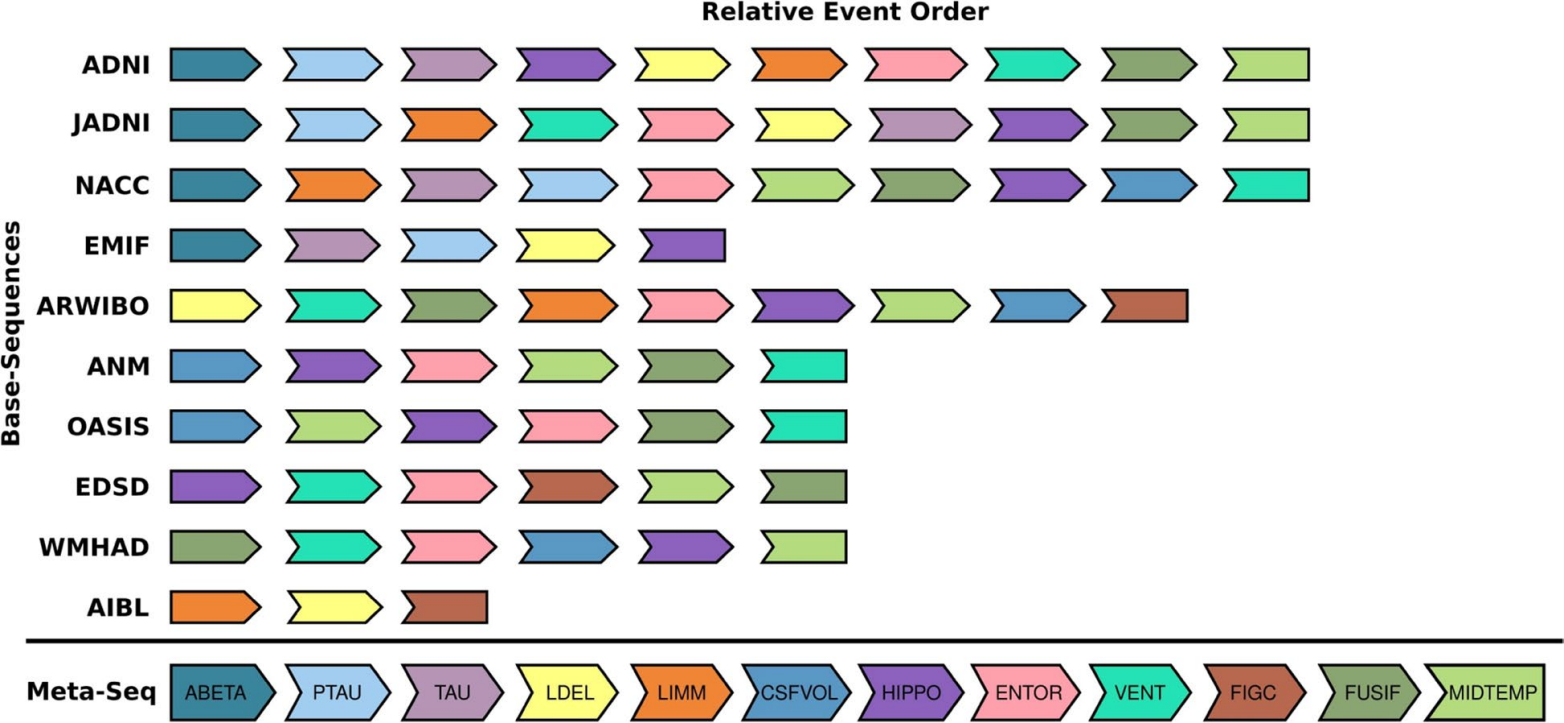
All ML base sequences from the ten investigated cohorts and the resulting meta-sequence. Due to only partially overlapping lists, the determining factor for an event’s position in the meta-sequence was not its absolute position in each base sequence (i.e., rank 1, 2, …, 11), but its relative position to other biomarkers in the same sequence (e.g., ABETA commonly places before MMSE when they were assessed together; thus, it appears before MMSE in the meta-sequence)

In the meta-sequence generated based on each cohort’s ML sequence (Fig. 2), ABETA was ranked first, followed by PTAU and TAU. The latter were again closely linked and seemingly interchangeable given their ambiguous positioning across the base sequences. In positions four and five, LDEL and LIMM followed respectively, two clinical assessments measuring memory impairment. Next, the volume of CSF in the brain was positioned in the meta-sequence. The later event ranks were covered by MRI markers of brain volume, starting with the temporal lobe (e.g., hippocampus and entorhinal cortex) and ending with the ventricles. The previously described ambiguity in the order of MRI regions is not reflected in the ML-based meta-sequence because the algorithm considers only the ranks, and not the uncertainty estimated by the individual EBMs. However, it seems sensible to consider MRI events as fairly interchangeable in the meta-model. FIGC, an assessment of visual function, positioned before FUSIF and MIDTEMP near the end of the sequence, yet its position with respect to those two variables remained rather indefinite across the base sequences in which it was assessed (ARWIBO, AIBL, EDSD).

**Fig. 3:**
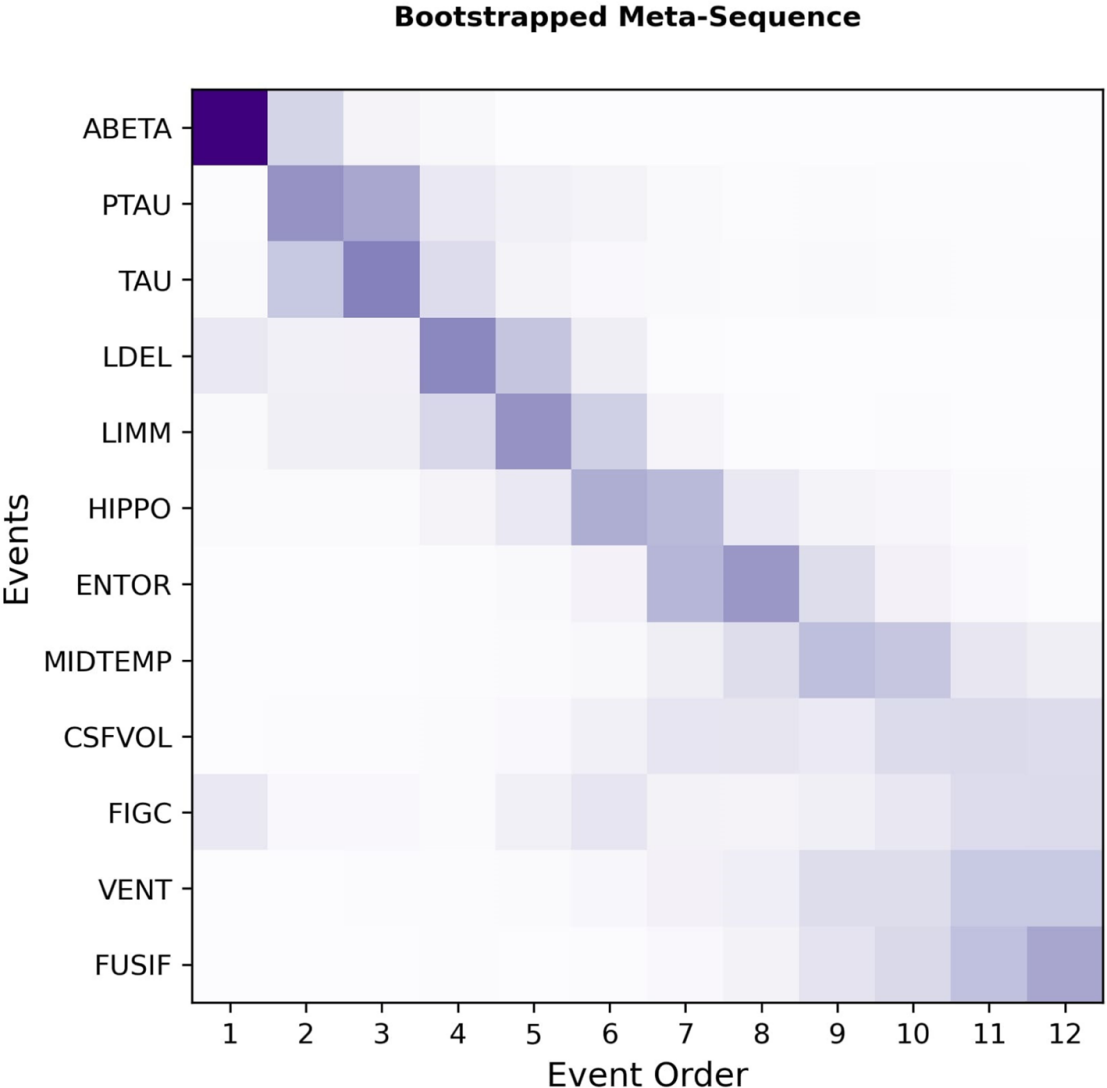
Bootstrapped meta-sequence generated from 500 samples of the base sequences of the 10 cohorts. Event order 1 corresponds to the first position in the sequence. The shading of squares indicates the positional probability with darker shades corresponding to higher probabilities

The consensus meta-sequence generated using the bootstrapping approach resembled the ML meta-sequence closely (KTC between both meta-sequences: 0.79; Fig. 3). Again, CSF markers placed first in the meta-sequence, were followed by cognitive assessments, and MRI events started with the temporal lobe and further progressed with the ventricles. The main difference to the ML-based meta-sequence, as well as the major region of model uncertainty, was again found among the MRI variables. This further underlined the impression that the MRI events were fairly interchangeable and probably occurred in close temporal proximity. The highest ambiguity was in the positioning of FIGC which showed a slight tendency towards the last ranks. The average KTC across all bootstrapped meta-sequences was 0.5 ± 0.20, with the highest discordance found among the MRI modality.

**Fig. 4:**
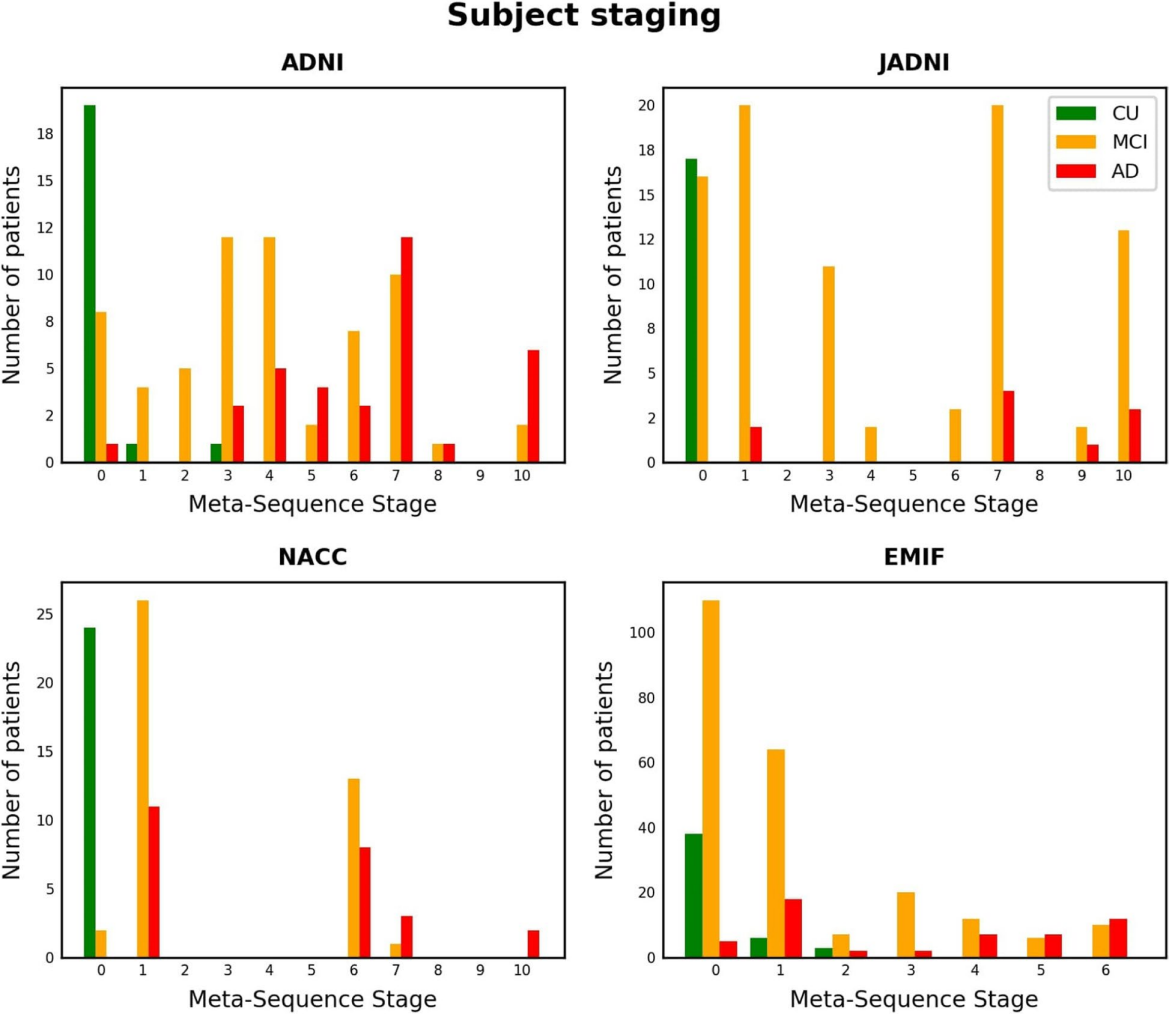
Number of subjects from each diagnostic group per meta-sequence stage. Each step along the *x*-axis corresponds to the occurrence of a new biomarker abnormality event. Stage 0 corresponds to no event occurrence while the last stage implies abnormality of all variables. Events are ordered according to the bootstrapped meta-sequence, always considering only variables in common between the measurements available in the respective cohort and the meta-sequence

Staging the patients of cohorts with available CSF, MRI, and cognitive scores (i.e., ADNI, JADI, NACC, EMIF) revealed a consistent pattern across them (Fig. 4). For all cohorts, the vast majority of CU subjects were assigned to the first stage which corresponds to no event occurrences. As expected, MCI patients were largely staged between CU subjects and AD patients with some overlap in both directions. This suggests that these subjects experienced CSF marker abnormalities and some cognitive symptoms. Finally, the majority of AD patients were assigned to the last stages, indicating their abnormality along CSF markers, cognitive performance, and brain region atrophy.

## Discussion

In this work, we used EBMs to investigate AD progression across ten independent cohort studies by evaluating the concurrence of their individually derived event sequences. Furthermore, we proposed an algorithm to combine event sequences estimated from partially overlapping, and thus complementary, sets of variables into a single meta-sequence describing AD progression more comprehensively. Finally, we applied said algorithm on the ten event sequences to estimate a meta-sequence comprising 13 AD variables spanning CSF biomarkers, MRI measures, and clinical assessments of cognitive and functional performance.

### Consistent trends across cohorts’ event sequences

The derived event sequences proved to be broadly consistent across cohorts, with the most notable variability in the ordering of MRI brain volume events. This could be caused by (1) distinct statistical biases of the cohorts for example introduced through specific recruitment criteria [21], (2) distinct prevalence of AD disease progression subtypes that follow different disease mechanisms [38–40], or (3) mixed neuropathologies.

Inclusion and exclusion criteria of a study shape the demographic compositions of its cohort and thus can directly affect the data-driven disease progression patterns (Table S3). For instance, ADNI held a higher proportion of APOE4 carriers compared to JADNI. Given that it has been repeatedly reported that early TAU depositioning is more prominent in APOE4 carriers [41–43], this difference might explain the earlier positioning of TAU in ADNI’s sequence opposed to its relatively lower rank in JADNI’s.

Previously, for example, two empirically determined AD progression subtypes called “hippocampal-sparing” and “limbic-predominant” were described and associated with distinct patterns of brain atrophy [38, 44]. While structural changes in the brain start with atrophy in the medial temporal lobe (e.g., entorhinal and hippocampus) for the limbic-predominant subtype, the brain deterioration in the hippocampal-sparing subtype begins with atrophy of the frontal cortex and with the enlargement of ventricles [44]. Given their respective event sequences, this could indicate that OASIS, ADNI, and NACC might have included more patients expressing the limbic-predominant subtype, while the hippocampal-sparing subtype was more dominant among patients from ARWIBO and JADNI.

We observed that CSF biomarkers placed first in all cohorts which measured them. This finding is in concordance with previous biomarker studies that observed the occurrence of both ABETA accumulation and brain atrophy before global cognitive decline [45–48].

Autopsies of AD patients have shown that AD pathology hardly appears in isolation and that patients often suffer from a mixture of brain pathologies [49]. While most studies aim to exclude patients affected by other cognitive diseases, an AD clinical diagnosis is still mainly symptom driven and misclassification errors are possible.

### Meta-sequence combines heterogeneous event sequences from multiple cohorts

A particular strength of our meta-sequence algorithm is that it works agnostic towards the differences in variable value representations exhibited across cohorts. A direct comparison of the provided data values often remains challenging without introducing statistical biases since studies differ, for example, in their data collection procedures, employed imaging machinery, and used assays. Using our approach, such semantically equivalent but statistically heterogeneous information can be combined as all computations are performed solely on the base sequences and thus potential across-cohort-biases due to value representations are avoided.

The biggest advantage of the bootstrapping approach compared to ML sequence-based one is that it allows for uncertainty quantification. However, bootstrapped EBM sequences tend to display a substantially higher positional variance (i.e., “fuzziness”) than ML derived ones (for an example, see Firth et al. Figures 1 and 2 [35]). Comparing our ML-based meta-sequence to the bootstrapping-based meta-sequence revealed high similarity between them. Observed differences seemed to be within variational limits expressed in the bootstrapped meta-sequence and mainly affected MRI variables.

### Generated meta-sequence resembles AD pathology

One possibility to validate the derived meta-sequence was to evaluate its concordance with previous findings describing the temporal relationship between smaller subgroups of variables.

The ordering of CSF biomarkers discovered in previous EBM studies supported our observations in the meta-sequence (ABETA followed by PTAU and TAU) [15]. Our findings were also in line with a recent study [50] which demonstrated that TAU and PTAU become abnormal after ABETA and that their abnormality occurred in close temporal relationship with cognitive decline. The latter was also in concordance with our findings; however, the cognitive assessments we investigated (i.e., LDEL and LIMM) were not directly included in the referenced study. Furthermore, there is a well-established association between cognitive decline and ABETA abnormality and abundant evidence that changes in cognition typically occur after abnormalities related to CSF biomarkers [45, 50, 51].

Our observation that memory function showed abnormality before brain volumes agrees with previous studies which suggested that individual-level brain atrophy rates (not assessed in our study) precede cognitive events; however, MRI-derived brain volumes become abnormal afterwards [15].

In our meta-sequences, changes in MRI biomarkers were ranked after cognitive decline. In agreement with this, for example, Hadjichrysanthou et al. reported that changes in MRI markers appear in close succession with memory decline [52]. Also, the positioning of MRI variables with respect to CSF markers was concordant with previous observations where significant correlations between CSF biomarkers and temporal lobe atrophy were found [53–55]. These studies argue that increases of TAU and PTAU are attributable to the deposition of neurofibrillary tangles in the temporal lobe, including the hippocampus and entorhinal cortex, which we found to be the first brain region volumes turning abnormal. Furthermore, elevated CSF biomarkers predicted future brain atrophy in these regions (i.e., CSF biomarkers became abnormal before brain volumes).

In concordance with the relative positioning of MRI biomarkers in the meta-sequence, various studies have shown that volumetric changes start with the temporal lobe areas, including the hippocampus which preceded the abnormality of the entorhinal cortex, fusiform, and middle temporal, and further proceed to other brain regions such as the ventricles [56–59].

Finally, in agreement with a previous study [60–63] in which visual memory dysfunction was identified as one of the last stages in AD progression, the FIGC test was ranked among the end of the sequences. The fact that it was positioned after the enlargement of ventricles is in agreement with experimental evidence that changes in the ventricles may precede a deficit in visual memory function [64, 65]. Another EBM study [35] also suggested that visual processing becomes impaired after episodic memory in typical AD.

The conducted patient staging provided further evidence that the generated meta-sequence described a sensible cascade of AD progression: participants from the three diagnostic groups were distributed according to their disease severity with CU subjects being staged first, MCI patients spreading around the intermediate stages, and AD cases occupying the later stages of the sequence. Observing MCI subjects at stage 0 could be explained by CSF biomarker values and cognitive scores that were close to the probabilistic event threshold but did not yet exceed it and, consequently, the model considered them to be normal. The few AD cases that were staged early in the sequence were amyloid-negative subjects which potentially indicated their misclassification.

### Limitations

To build a robust meta-sequence, each variable had to be present in at least some of the base sequences to allow for meaningful distance calculations. Furthermore, the high amounts of missing data occurring when multiple data modalities are combined led to a substantial decrease of the number of available participants per study. This could have led to more noise in the EBM’s reference distributions. Additionally, modeling signals from heterogeneous data sources, such as AD cohort data, as some form of average bears the potential risk that the resulting average will resemble a rather artificial construct that cannot be observed in its specific form in the real world. However, the similarity among the base sequences as well as between base sequences and the final meta-sequence was quite high and our identified meta-sequences were highly concordant with results from both data-driven and experimental studies. Furthermore, the patient staging along the meta-sequence displayed a sensible distribution of CU, MCI, and AD subjects along the disease stages. Consequently, it is improbable that the presented meta-sequence represents such an artificial average. Finally, we want to highlight again that AD was considered primarily from a clinical perspective in all of our investigated cohort studies. As such, there is a chance that misdiagnosed patients were present in the cohorts and therefore included in this analysis as well.

## Conclusion

In the light of the reproducibility crisis, it becomes especially important that we look beyond single data resources, validate achieved results across multiple cohort studies, and constantly develop and evaluate data-driven methods. To this end, we revealed general consistency across data-driven event sequences derived from ten independent cohorts using EBMs. Here, only relatively minor differences in the ranking of the core features that were available in all ten cohorts were observed. In addition, our novel algorithm estimated a meta-sequence that exploits the additional information available in other variables unique to each study and thus could assemble an event sequence that is highly multimodal and more comprehensive than sequences built from single datasets. This is important for ensuring the transferability of models and results across AD (sub)populations and for improving our understanding of disease progression.

## Supplementary Information

**Additional file 1.**

## Data Availability

De-identified data used in preparation of this article were obtained from the Alzheimer’s Disease Neuroimaging Initiative (ADNI) (https://adni.loni.usc.edu), the Australian Imaging, Biomarker and Lifestyle Flagship Study of Ageing (AIBL) database (https://aibl.csiro.au/), the European Collaboration for the Discovery of Novel Biomarkers for Alzheimer’s Disease (AddNeuroMed) (https://www.synapse.org/#!Synapse:syn4988768), Alzheimer’s Disease Repository Without Borders (ARWIBO) (https://www.neugrid2.eu/), Open Access Series of Imaging Studies (OASIS) (https://www.neugrid2.eu/), White Matter Hyperintensities in Alzheimer’s Disease (WMH-AD) (https://www.neugrid2.eu/), European Diffusion Tensor Imaging Study in Dementia (EDSD) (https://www.neugrid2.eu/), National Alzheimer’s Coordinating Center (NACC) (https://naccdata.org/), Japanese Alzheimer’s Disease Neuroimaging Initiative (JADNI) (https://humandbs.biosciencedbc.jp/en/hum0043-v1), European Medical Information Framework for Alzheimer’s Disease Multimodal Biomarker Discovery (EMIF-AD MBD) (https://emif-catalogue.eu; http://www.emif.eu/about/emif-ad). The authors had no special access privileges others would not have to the data obtained from these resources.

## Abbreviations

ADNI: The Alzheimer’s Disease Neuroimaging Initiative
JADNI: Japanese Alzheimer’s Disease Neuroimaging Initiative
AIBL: The Australian Imaging, Biomarker Lifestyle Flagship Study of Ageing
NACC: The National Alzheimer’s Coordinating Center
ANM: AddNeuroMed
EMIF-1000: European Medical Information Framework
EDSD: European DTI Study on Dementia
ARWIBO: Alzheimer’s Disease Repository Without Borders
OASIS: Open Access Series of Imaging Studies
WMHAD: White Matter Hyperintensities in Alzheimer’s Disease
CDRSB: Clinical Dementia Rating Sum of Boxes
NPI: Neuropsychiatric Inventory
LDEL: Logical Memory - Delayed Recall Total Number of Story Units Recalled
ADAS13: Alzheimer’s Disease Assessment Scale (13-items)
ADAS11: Alzheimer’s Disease Assessment Scale (11-items)
MMSE: Mini-Mental State Examination
LIMM: Logical Memory - Immediate Recall Total Number of Story Units Recalled
TRABS: Trail Making Test-B
DIGITS: Digit-Symbol Coding Test
LIDE: California Verbal Learning Test Delayed Raw Score
CATFLU: Category Fluency (animals - fruits/vegetables)
FIGC: Figure Copy
LIRE: California Verbal Learning Test Recall Raw Score
FIGR: Figure recall
STROOP: C/D Stroop Test Raw
STM: Short-Term Memory
LANGU: Language
ORIENT: Perceptual Orientation
MENMA: Mental Manipulation
ATTEN: Attention
CLKS: Clock Drawing Test Total Score
EXECUTIVE: Executive Memory
LICOR: Word List Learning Trial
BNTS: Boston Naming Test Score
WAIS: Digit Symbol Substitution Test
ABETA: Amyloid-β
TAU: Total Tau
PTAU: Phosphorylated Tau (p-Tau)
ENTOR: Entorhinal volume
HIPPO: Hippocampal volume
FUSIF: Fusiform volume
VENT: Ventricles volume
MIDTEPM: Middle temporal volume
CSFVOL: Accumulated CSF in the brain
FDG: Fluorodeoxyglucose positron emission tomography (FDG PET)
MRI: Magnetic resonance imaging
MCI: Mild cognitive impairment
AD: Alzheimer’s disease
CU: Cognitive unimpaired
KTC: Kendall’s tau rank correlations
EBM: Event-based model
CSF: Cerebrospinal fluid
