# Supplementary material for: Comparison and aggregation of event sequences across ten cohorts to describe the consensus biomarker evolution in Alzheimer’s disease
Source: Alzheimers Res Ther. 2022 Apr 20;14:55. doi: 10.1186/s13195-022-01001-y (PMC9020023; doi:10.1186/s13195-022-01001-y)
Supplement: Supplementary file 1 — Additional file 1. [file 13195_2022_1001_MOESM1_ESM.docx]

# Supplementary File

# Data preprocessing

While in some of the cohorts brain volumes were calculated as a sum of the two respective hemispheres, in others they were measured individually per hemisphere and thus we had to sum them up to make the measurements consistent across all cohorts. In addition, the brain region volumes of individual cohorts were normalized across the subjects based on their whole brain volume to correct the variations of head size among individuals by dividing the regional volumes through the intracranial volume.

## **Example of variable inclusion and relation to diminishing number of patients**

Below we provide an example to illustrate the decrease in sample size when integrating multimodal AD cohort data and considering only complete cases (ie. no missing data in any variable). We also excluded MCI patients in this example, as only CU and AD were the crucial diagnostic groups for fitting our EBMs. The example is based on a potential inclusion of amyloid PET. **Table S1** provides an overview about the stepwise decrease in participants available for analysis.

Out of the 10 cohorts we analyzed, only ADNI, EMIF, AIBL, and NACC reported measures of amyloid PET, already reducing the number of potentially analyzable cohorts to 4 out of 10. NACC only reported binary values (0, 1) which could not be modeled using our approach. Focusing only on our selected cognitive variables at baseline, ADNI had complete measurements for 229 CU and 183 AD participants, EMIF for 140 CU, 114 AD, and AIBL for 92 CU, 13 AD. Now, combining this data with MRI variables available in each cohort reduced the number of CU/AD to 204 / 130, 67 / 87, and no remaining participants, respectively. Further adding CSF measurements again decreased the sample size to 38 CU and 35 AD for ADNI, 47 CU and 53 AD for EMIF, and no remaining participants for AIBL.

| Cohort | ADNI | | EMIF | | AIBL | |
| --- | --- | --- | --- | --- | --- | --- |
| Diagnosis | CU | AD | CU | AD | CU | AD |
| Total | 813 | 389 | 230 | 184 | 803 | 181 |
| Cognitive Scores | 229 | 183 | 140 | 114 | 92 | 13 |
| + MRI | 204 | 130 | 67 | 87 | 0 | 0 |
| + CSF | 38 | 35 | 47 | 53 | 0 | 0 |
| + Amyloid PET | 0 | 0 | 47 | 53 | 0 | 0 |

**Table S1:** Decrease in sample size when aiming for a multimodel analysis of amyloid PET measurements and stepwise adding additional modalities while recording only complete cases (ie. no missing values in any selected variable).

# Event-based models

While prior versions of event-based models (EBMs) mainly integrated parametric mixture models (i.e. Gaussian mixture models; GMM) [1-3], we leveraged its latest installment which incorporates nonparametric mixture models by employing a kernel density estimation (KDE) to determine the probability density function [4]. KDE estimates the probability density of independent and identically distributed samples (x_1_, x_2_, …, x_n_) drawn from a distribution with an unknown density by


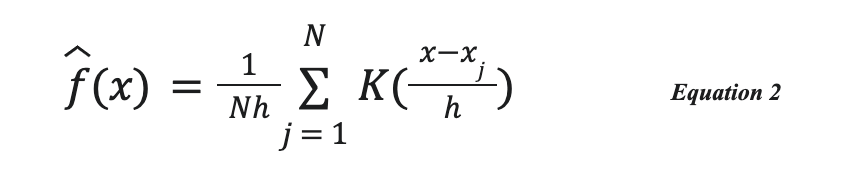


where *K* and *h* are kernel function and bandwidth, respectively **[5]**. The kernel we used was Gaussian and the bandwidth for estimating the components of the mixture models was determined by Scott's normal reference rule **[6]**.

# Meta-sequence generating algorithm

Our proposed method for generating a meta-sequence from multiple, complementary base sequences represents an algorithm addressing the rank aggregation of partial lists **[7]**. It essentially solves an optimization problem (i.e., finding the meta-sequence with the smallest distance to all base-sequences) by combining an exhaustive search for the initial $k$-length starting sequence with a greedy search procedure adding missing variables into the respective position in the sequence where the average distance of the altered sequence to all base-sequences remains minimal. The reason for combining these two steps lies in the combinatorial explosion of the search space (i.e., the set of all theoretically possible meta-sequences) when including an increasing number of variables. Therefore, an exhaustive search is often computationally infeasible and heuristic approaches have to be considered instead. In such cases, rank aggregation approaches often rely on Monte Carlo sampling to test a set of random meta-sequences and then opt for the one with the lowest distance **[8]**. However, we found that these approaches often end with suboptimal meta-sequences for large search spaces and that the proposed approach mixing an exhaustive search for a starting sequence with subsequent greedy refinements leads to more robust and plausible results, both biologically and in comparison to the base sequences. In theory, multiple meta-sequences could be identified that share the minimum distance to the base sequences (Figure S1). The python code for running this algorithm can be found under (<https://github.com/sepehrgolriz/EBM-MultiCohort>).

The bootstrapping-based version of our algorithm follows the same logic, however, the process is repeated *b* times, where the base sequences are determined based on a bootstrap sample of each respective cohort (ie. a sampling with replacement of the cohort of equal size as the original data).


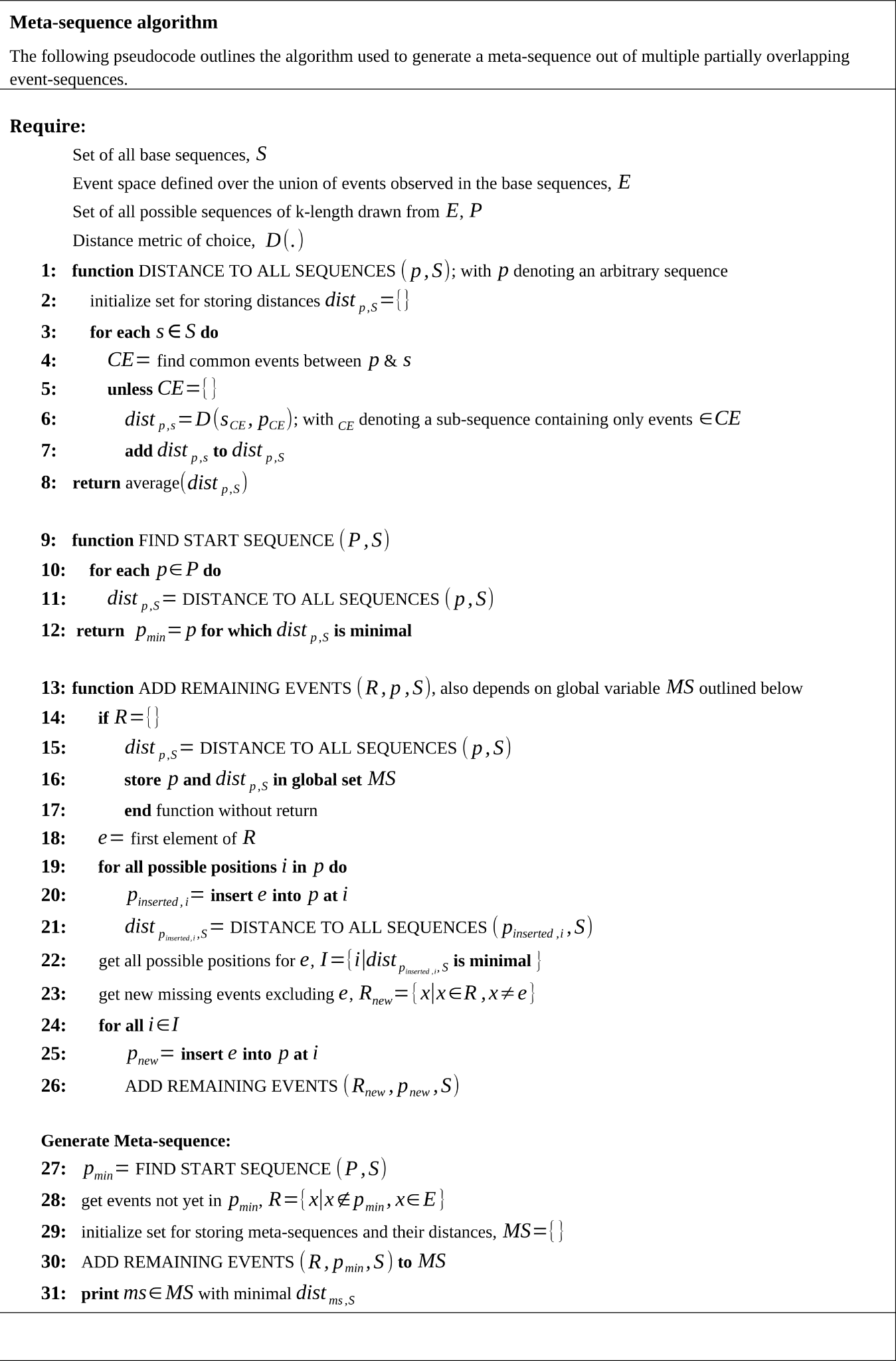


**Figure S1.** Proposed algorithm for determining a meta-sequence from multiple potentially only partially overlapping base sequences. Line 23: All possible orderings in which to add the remaining events are tested.

### Handling partially overlapping lists

Distance calculations have to be performed in the same mathematical space which, in this case, is defined by the variables in the sequences to be compared. Calculating the distance between two sequences which share the same variables is therefore straight forward. However, since individual base-sequences are often only partially overlapping, such distance calculations are impeded. There are two solutions for this problem: 1) penalizing the absence of variables in either sequence such that the distance increases with a higher number of uncommon variables, or 2) ignore variables that are only present in one of the sequences when calculating the distance. In the context of clinical cohort data, whether a specific variable was assessed depends on the study’s goals and funding and, as such, its absence does seldomly hold biological meaning. Therefore, in this case, penalizing the absence of variables would bias the constructed meta-sequence.

**Distance metrics**

Depending on the focus of the study, different distance metrics can be used in the proposed algorithm. Intuitive choices are Spearman’s footrule distance or Kendall's tau. The former takes the magnitude of the rank differences into account, while the latter is only counting how many rank discrepancies are found between two compared sequences, ignoring their specific position. A decision on which metric should be used depends on the emphasis of the study. In this study, we used Spearman’s footrule distance because it takes the absolute difference in positions of variables into account which should be informative in our biomedical context.

# Patient staging

$argmax_{j} Pr(X_{j}|MS,d)=argmax_{j} P(d)\sum_{m = 0}^{M} \{\prod_{i = 1}^{m} Pr(x_{ij}|E_{i})\prod_{i=m+1}^{M} Pr(x_{ij}|\neg E_{i})$ Equation 3

Pr$(x_{ij}|E_{i})$ and Pr($x_{ij}|\neg E_{i}$) denote the probability of observing the value of $x$ given that event $E_{i}$ did, or did not, occurred, respectively. It is assumed that the probability of being at stage *d* is uniform. The final assignment of a particular subject to a certain stage *d* remains a probabilistic assignment and is not a definite description that this participant is exactly at that stage in the disease cascade.

# Supplementary Table

| **Cohort** | **Memory** | **Executive** | **Language** | **Visuospatial** | **Global cognitive** |
| --- | --- | --- | --- | --- | --- |
| ADNI | LIMM  LDEL | DIGIT  TRABS | - | - | ADAS11  ADAS13 |
| JADNI | LIMM  LDEL | DIGIT  TRABS | - | - | ADAS11  ADAS13 |
| NACC | LIMM | WAIS | BNTS  CATFLU | - | - |
| AILB | LIMM  LDEL | WAIS  STROOP | LIRE  LIDE  LICOR  CATFLU | FIGC FIGR | - |
| EMIF | LIMM  LDEL | EXECUTIVE | LANG | - | - |
| ANM | - | - | - | - | - |
| ARWIBO | LIMM  LDEL | - | - | FIGC | - |
| OASIS | - | - | - | - | - |
| EDSD | - | - | LIRE  LICOR  BNTS | FIGC FIGR  CLKS | - |
| WMAHD | STM | - | CATFLU | CLKS | - |

**Table S2.** Cohort-specific cognitive tests composing each cognitive domain.

| **Cohort** | **Variables** | Years of education (mean$\pm$std) | Age (mean$\pm$std) | APOE4% (at least one e4 allele) | Female% |
| --- | --- | --- | --- | --- | --- |
| ADNI | CU | 16 ± 2 | 74 ± 5 | 31 | 32 |
|  | MCI | 16 ± 2 | 73 ± 7 | 62 | 32 |
|  | AD | 15 ± 2 | 75 ± 7 | 79 | 40 |
|  | Total | 15 ± 2 | 74 ± 6 | 58 | 33 |
| JADNI | CU | 14 ± 4 | 69 ± 6 | 30 | 56 |
|  | MCI | 13 ± 4 | 73 ± 5 | 51 | 54 |
|  | AD | 12 ± 2 | 74 ± 3 | 62 | 55 |
|  | Total | 13 ± 3 | 72 ± 6 | 47 | 55 |
| ARWIBO | CU | 8 ± 2 | 52 ± 7 | 20 | 57 |
|  | MCI | 6 ± 4 | 71 ± 7 | 39 | 64 |
|  | AD | 5 ± 3 | 71 ± 8 | 42 | 81 |
|  | Total | 6 ± 2 | 64 ± 7 | 33 | 67 |
| OASIS | CU | 6 ± 2 | 52 ± 12 | - | 71 |
|  | MCI | 5 ± 2 | 74 ± 6 | - | 55 |
|  | AD | 3 ± 2 | 78 ± 4 | - | 66 |
|  | Total | 5 ± 1 | 68 ± 9 | - | 64 |
| EMIF | CU | 13 ± 5 | 69 ± 7 | 48 | 51 |
|  | MCI | 11 ± 5 | 68 ± 7 | 51 | 49 |
|  | AD | 10 ± 5 | 66 ± 7 | 52 | 50 |
|  | Total | 11 ± 7 | 67 ± 7 | 51 | 50 |
| ANM | CU | 10 ± 3 | 75 ± 4 | 24 | 59 |
|  | MCI | 7 ± 3 | 78 ± 7 | 38 | 54 |
|  | AD | 7 ± 3 | 79 ± 5 | 57 | 62 |
|  | Total | 8 ± 3 | 77 ± 6 | 39 | 58 |
| AIBL | CU | 12 ± 3 | - | 23 | 59 |
|  | MCI | 12 ± 7 | - | 52 | 48 |
|  | AD | 12 ± 2 | - | 52 | 48 |
|  | Total | 12 ± 2 | - | 42 | 51 |
| EDSD | CU | 13 ± 5 | 69 ± 3 | 31 | 51 |
|  | MCI | 11 ± 3 | 71 ± 5 | 47 | 43 |
|  | AD | 11 ± 5 | 73 ± 5 | 56 | 51 |
|  | Total | 12 ± 3 | 72 ± 6 | 44 | 48 |
| WMHAD | CU | 8 ± 2 | 73 ± 4 | 40 | 35 |
|  | MCI | 8 ± 2 | 77 ± 6 | 60 | 50 |
|  | AD | 8 ± 2 | 77 ± 6 | 50 | 80 |
|  | Total | 8 ± 2 | 75 ± 6 | 50 | 55 |
| NACC | CU | 16 ± 5 | 75 ± 4 | 38 | 71 |
|  | MCI | 16 ± 4 | 73 ± 4 | 47 | 40 |
|  | AD | 13 ± 5 | 73 ± 4 | 61 | 42 |
|  | Total | 16 ± 2 | 63 ± 4 | 48 | 51 |

**Table S3.** The table above summarizes the demographic characteristics as well as the total number of participants in each diagnostic group for the investigated cohort datasets. CU: Cognitively unimpaired. MCI: Mild cognitive impairment. AD: Alzheimer’s disease. Age: The average age of participants in each dataset. Years of Education: The average years of education of participants in each dataset. Female %: The percentage of female participants within each dataset. APOE4 Positive %: the percentage of participants with at least 1 APOE e4 allele. SD: Standard deviation. The years of education listed for OASIS participants seems irritatingly low, however, we found those values listed in the data.

|  | **AIBL** | **JADNI** | **ANM** | **WMHAD** | **ARWIBO** | **EMIF** | **OASIS** | **ADNI** | **EDSD** | **NACC** |
| --- | --- | --- | --- | --- | --- | --- | --- | --- | --- | --- |
| **AIBL** | 1 | - | - | - | 0.81 | - | - | - | 0.81 | 0.71 |
| **JADNI** | - | 1 | 0.78 | 0.73 | 0.60 | 0.801 | 0.73 | 0.72 | 0.78 | 0.86 |
| **ANM** | - | 0.78 | 1 | 0.82 | 0.85 | - | 0.70 | 0.90 | 0.90 | 0.62 |
| **WMHAD** | - | 0.73 | 0.82 | 1 | 0.81 | - | 0.73 | 0.90 | 0.91 | 0.67 |
| **ARWIBO** | 0.81 | 0.60 | 0.85 | 0.81 | 1 | 1 | 0.72 | 0.78 | 0.62 | 0.55 |
| **EMIF** | - | 0.80 | - | - | 1 | 1 | - | 0.85 | - | 0.90 |
| **OASIS** | - | 0.73 | 0.70 | 0.73 | 0.72 | - | 1 | 0.69 | 0.76 | 0.87 |
| **ADNI** | - | 0.72 | 0.90 | 0.90 | 0.78 | 0.84 | 0.69 | 1 | 0.90 | 0.73 |
| **EDSD** | 0.81 | 0.78 | 0.90 | 0.91 | 0.62 | - | 0.76 | 0.90 | 1 | 0.81 |
| **NACC** | 0.71 | 0.86 | 0.62 | 0.68 | 0.55 | 0.91 | 0.87 | 0.73 | 0.81 | 1 |

**Table S4.** Pairwise Kendall’s tau rank correlation coefficients

# Supplementary Figure

**
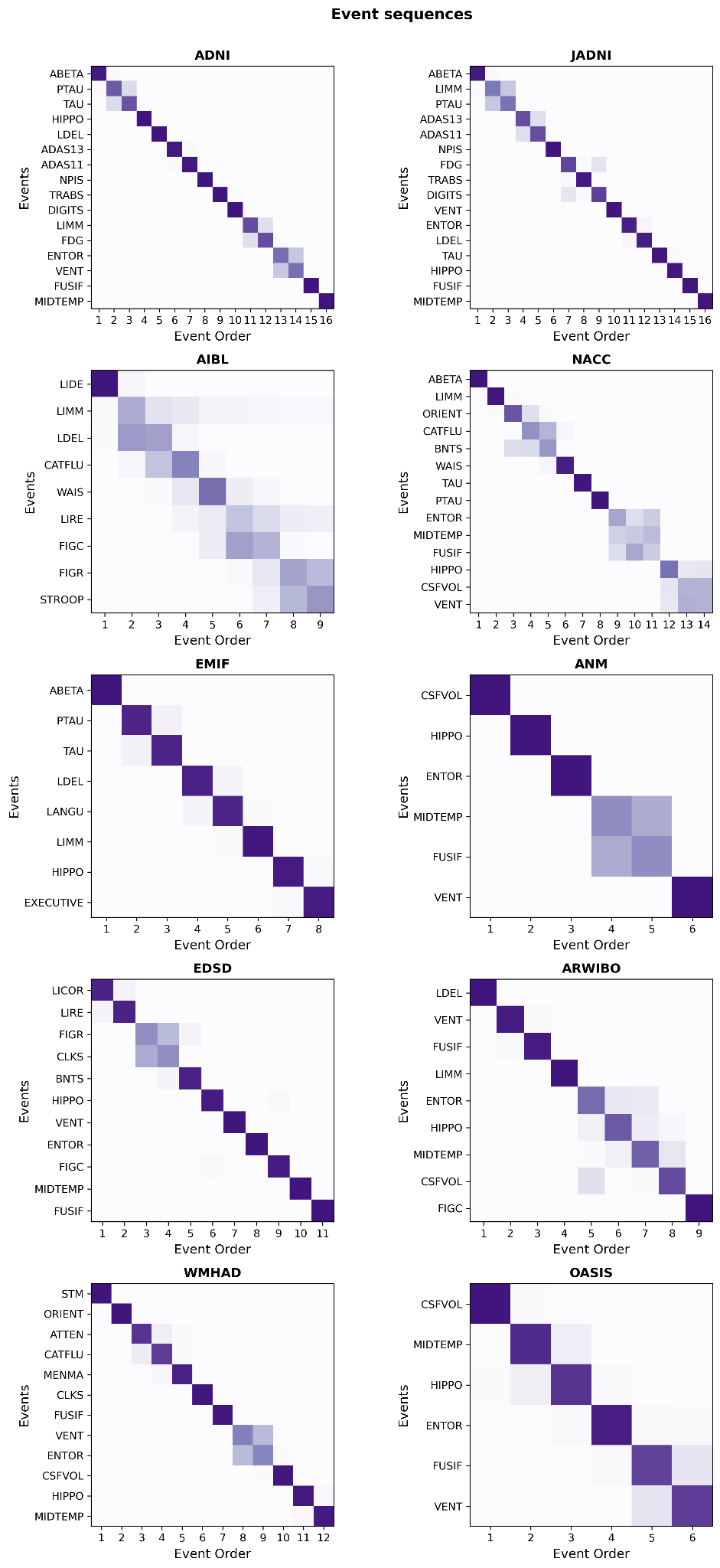
**

**Figure S2.** The original individual event sequences (independent y-axes) derived from the ten investigated cohorts. Event order 1 corresponds to the first position in the sequence. The shading of squares indicates the positional probability with darker shades corresponding to higher probabilities. The relative sizes of the squares do not encode any information.

**a)**


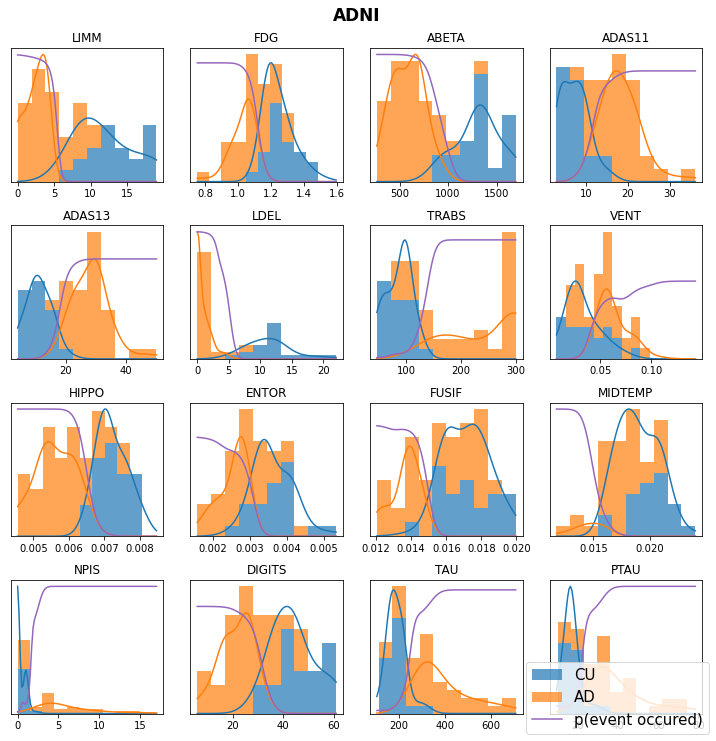


**b)**


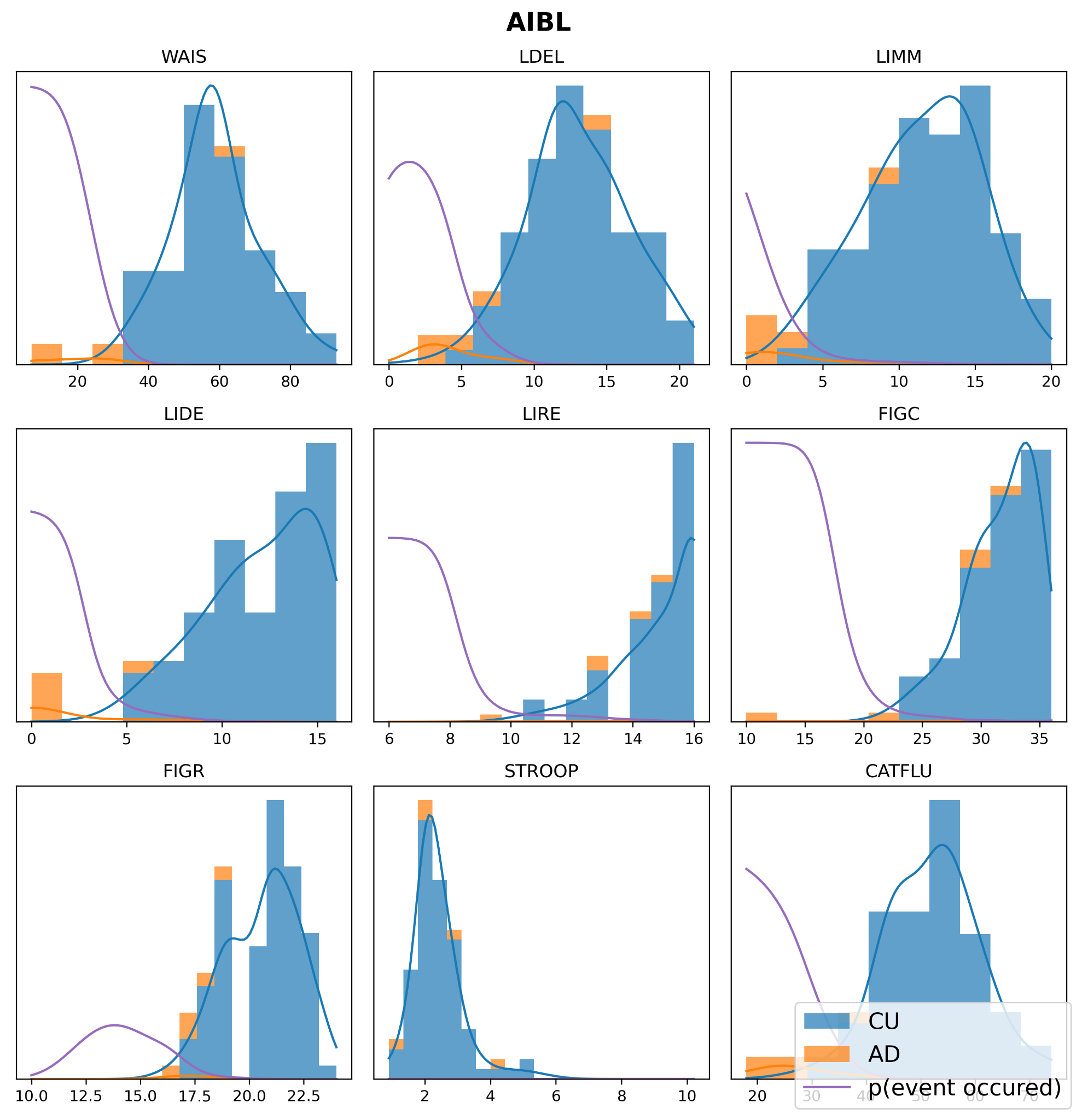


**c)**


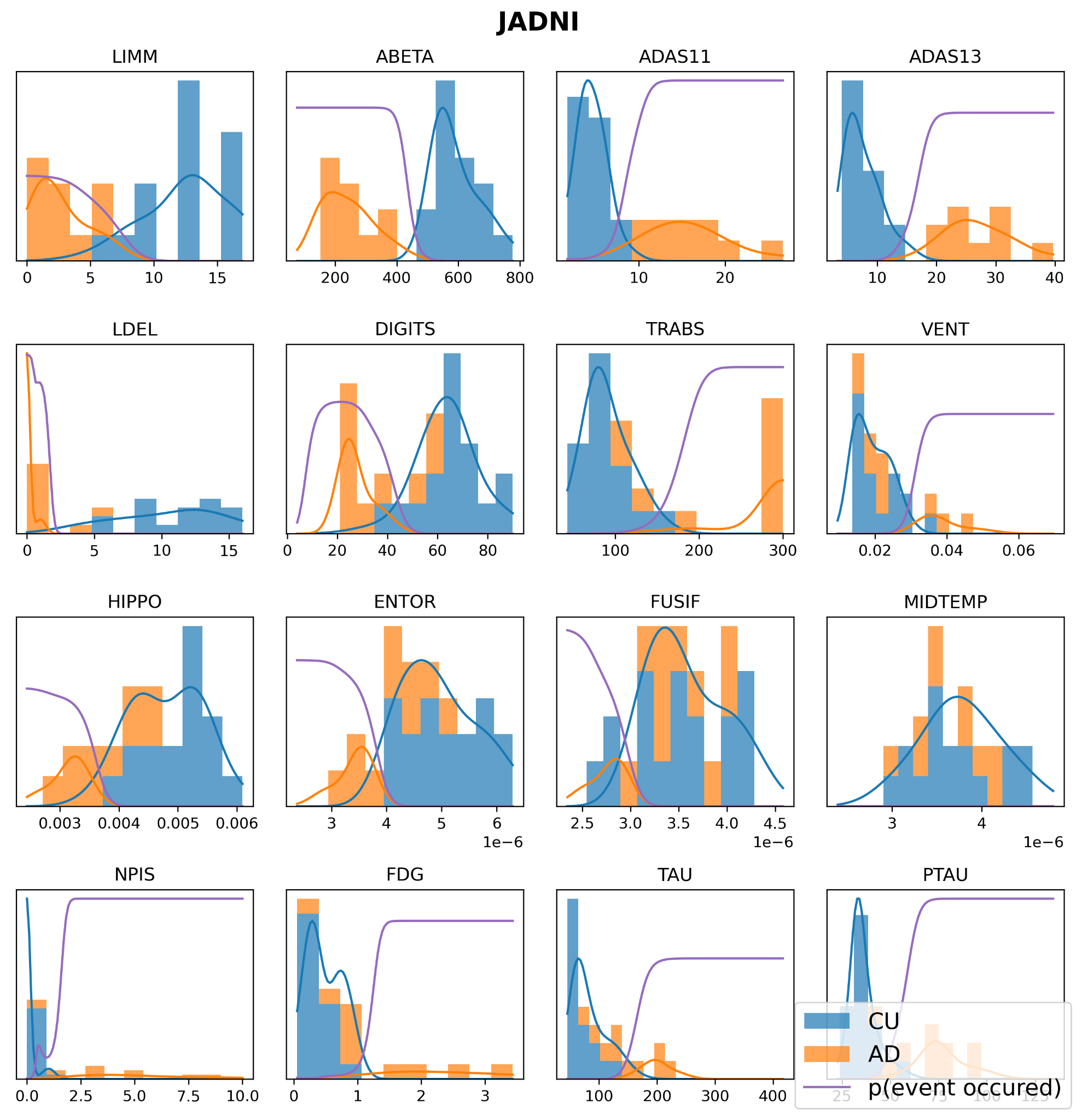


**d)**


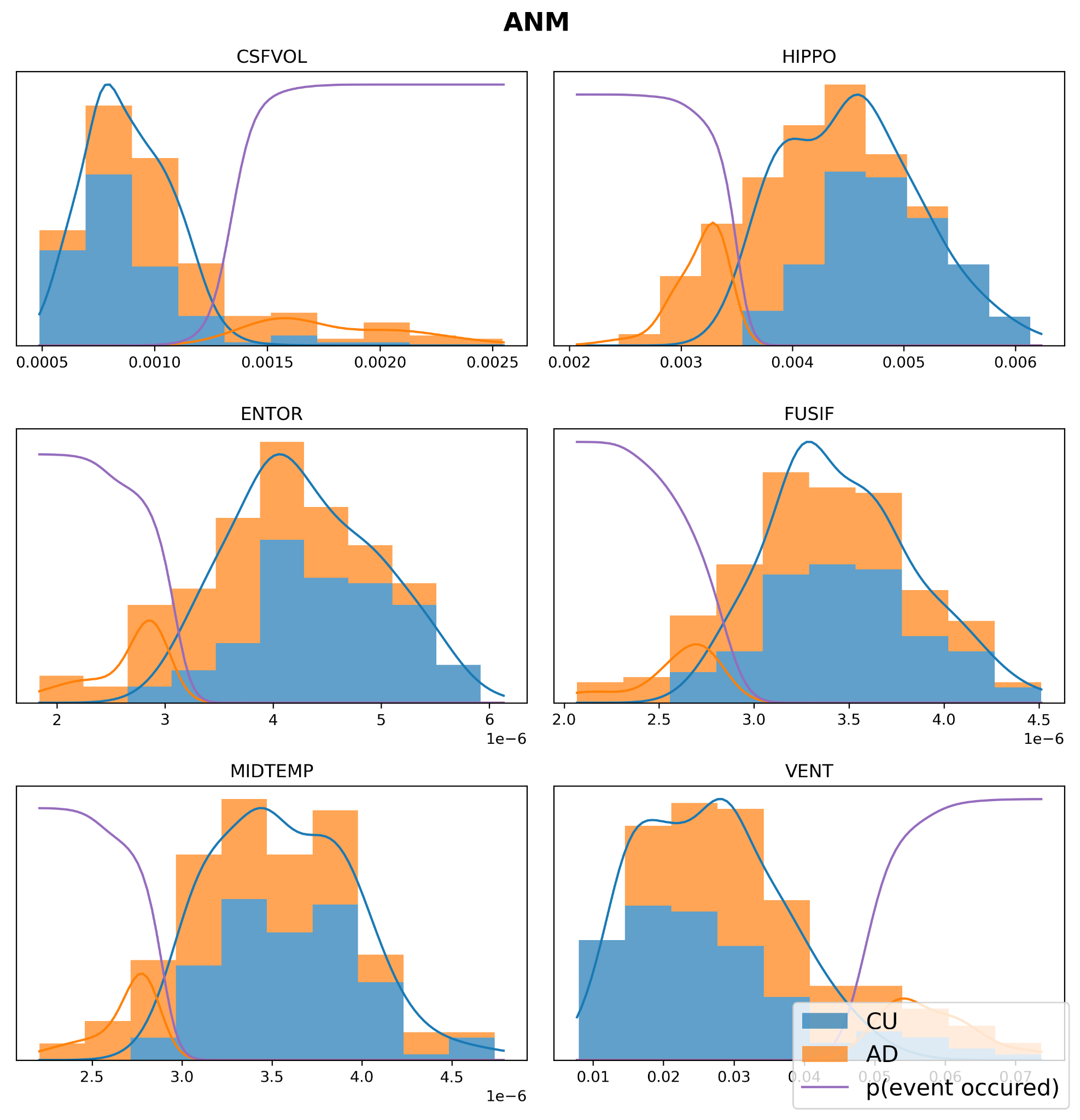


**e)**


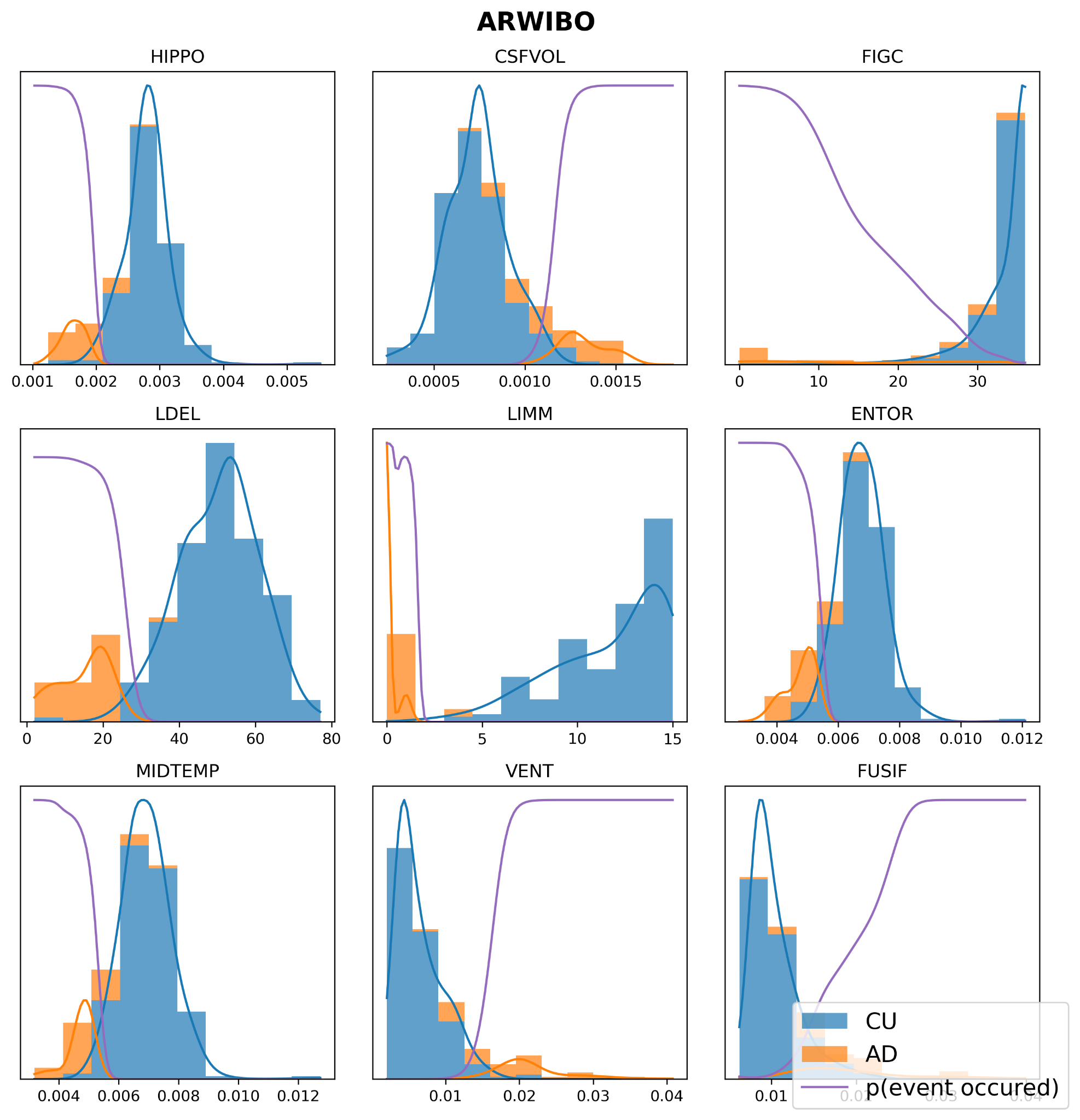


**f)**


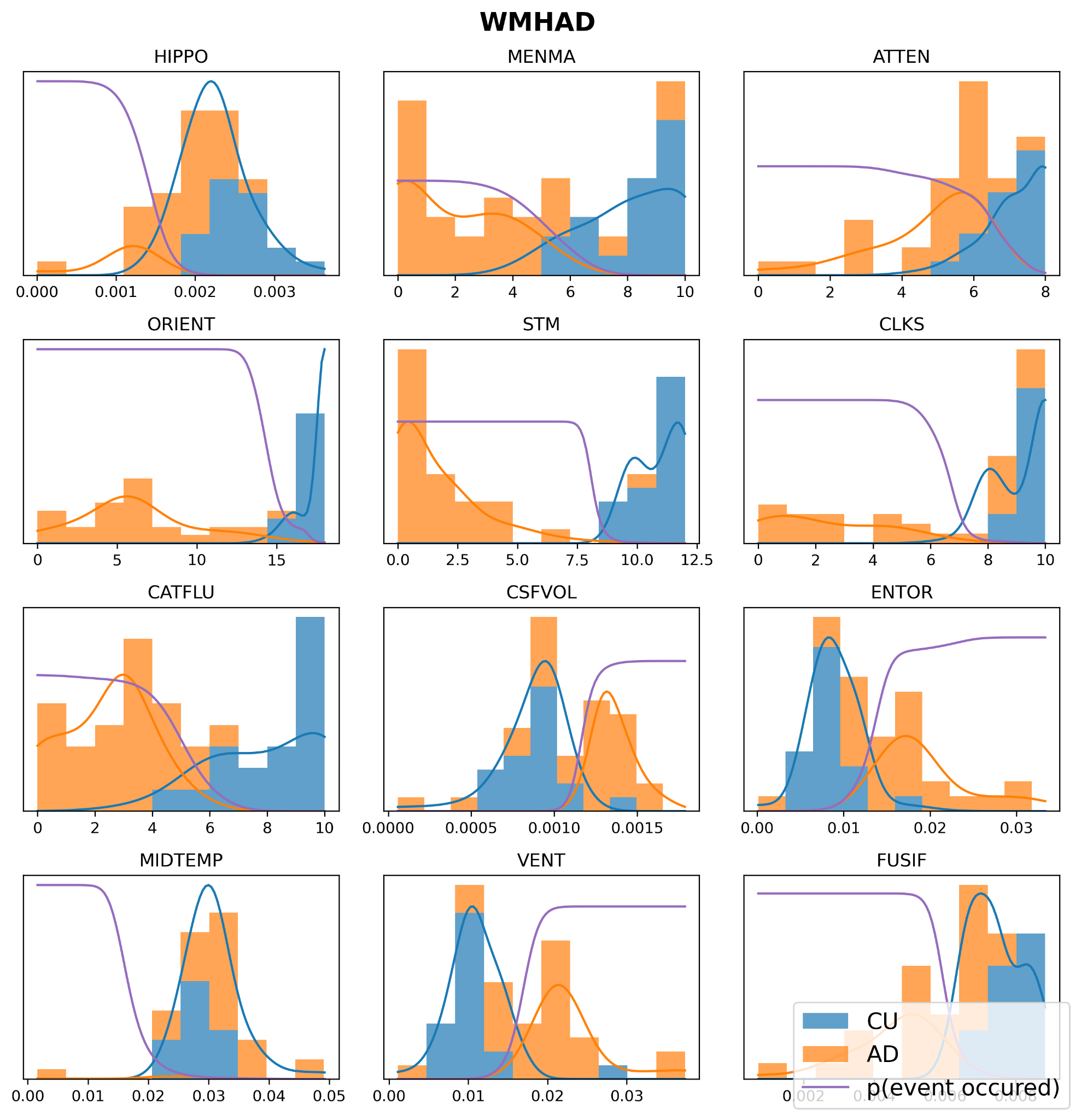


**g)**


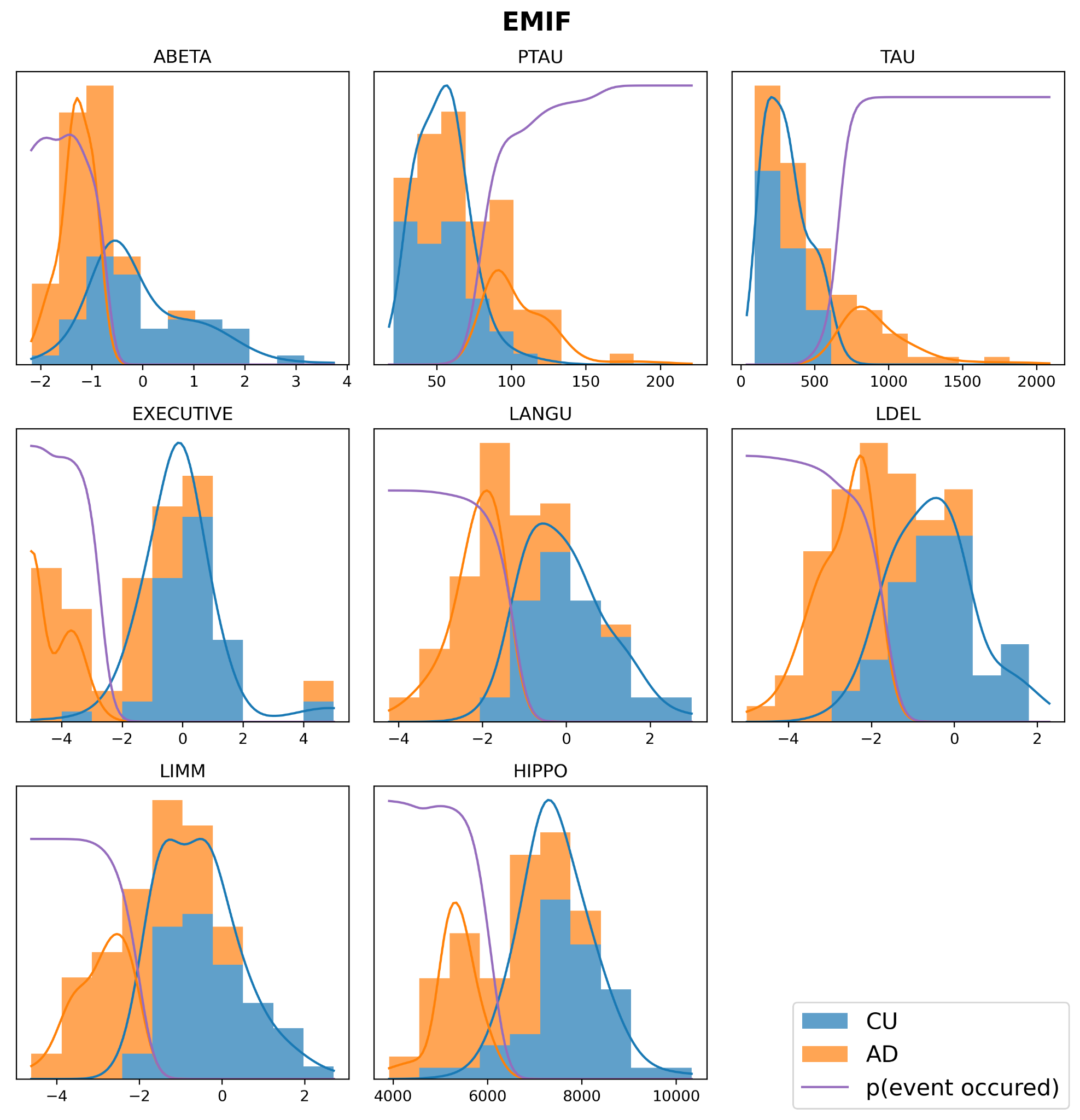


**h)**


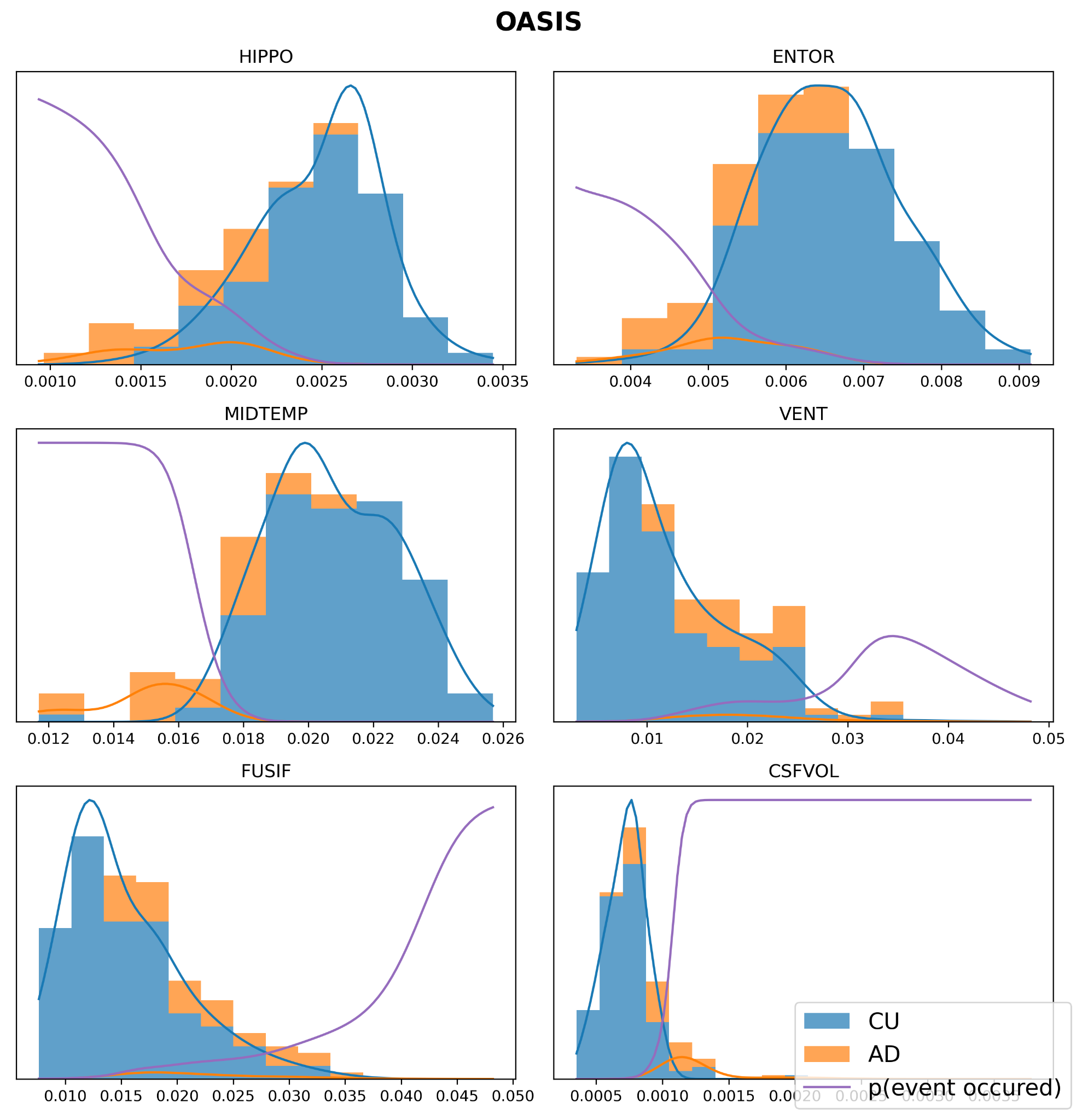


**i)**


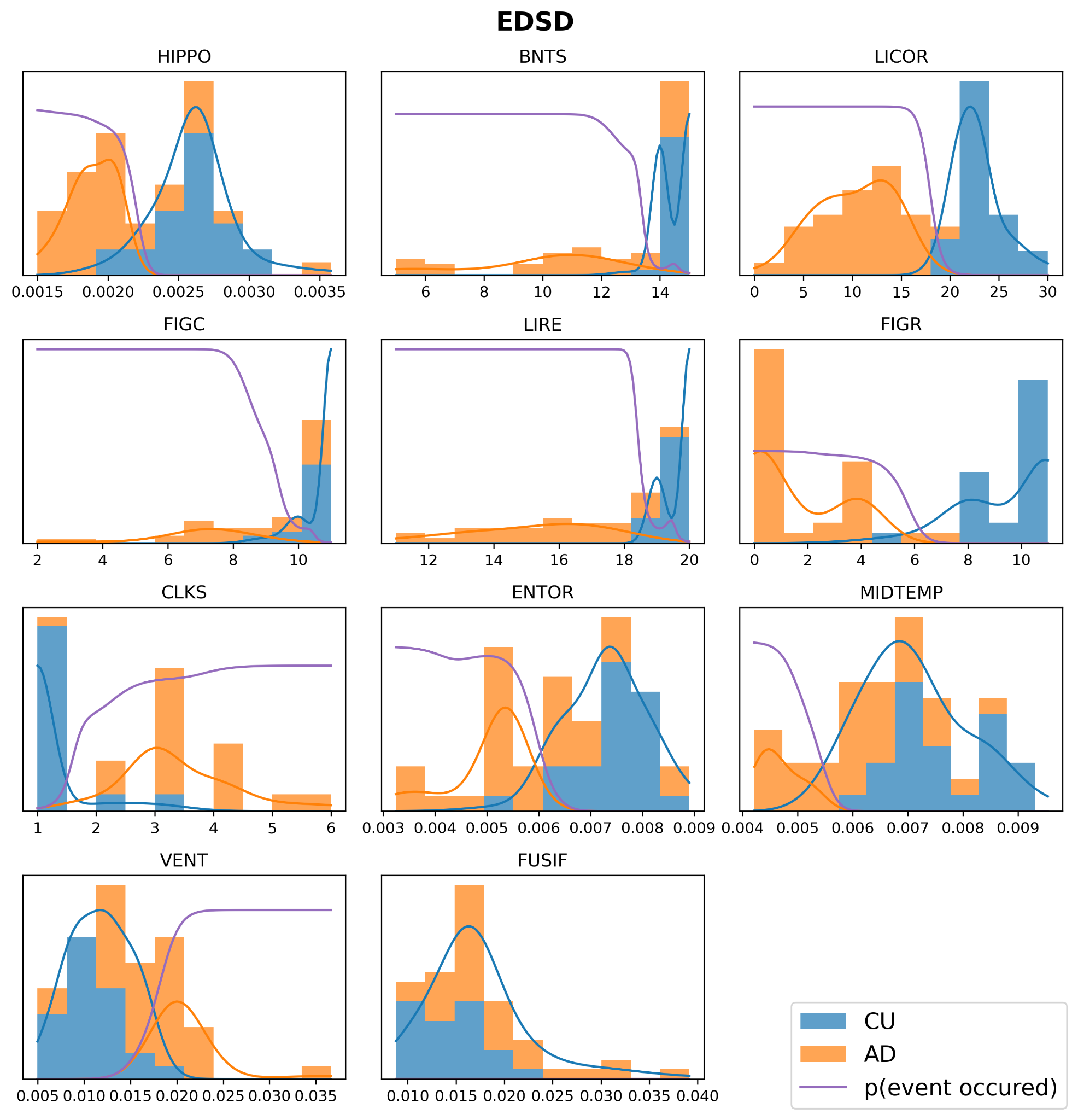


**j)**


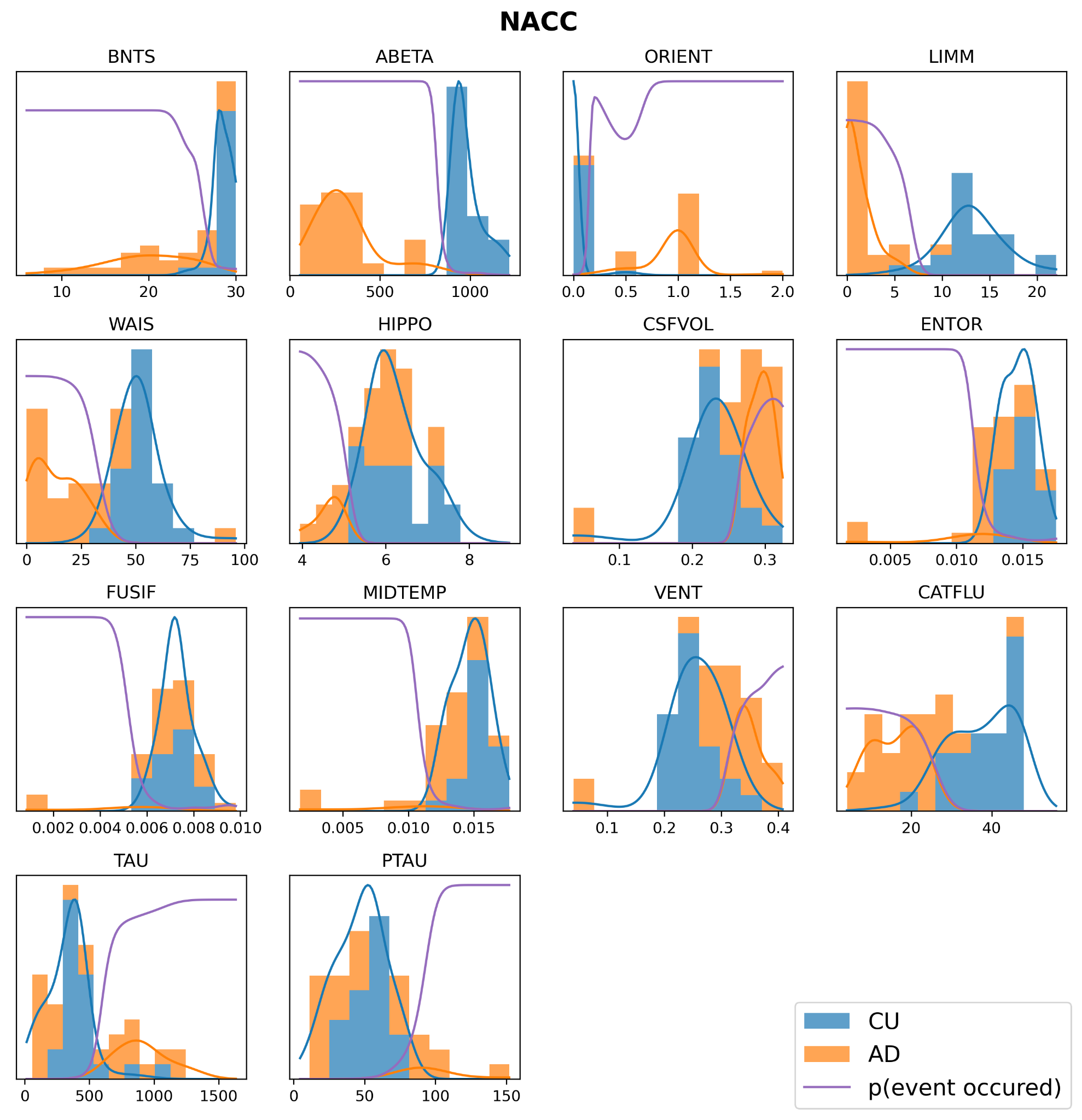


**Figure S3.** The derived mixture models for each cohort (a-j).

# References

1. Fonteijn, H. M., Modat, M., Clarkson, M. J., Barnes, J., Lehmann, M., Hobbs, N. Z., Scahill, R. I., *et al*. An event-based model for disease progression and its application in familial Alzheimer's disease and Huntington's disease. *NeuroImage* 2012, 60(3), 1880–1889. <https://doi.org/10.1016/j.neuroimage.2012.01.062>
2. Young, A. L., Oxtoby, N. P., Daga, P., Cash, D. M., Fox, N. C., Ourselin, S., Schott, J. M., *et al*. A data-driven model of biomarker changes in sporadic Alzheimer's disease. *Brain* 2014; 2564–2577. <https://doi.org/10.1093/brain/awu176>
3. Oxtoby, N. P., Young, A. L., Cash, D. M., Benzinger, T., Fagan, A. M., Morris, J. C., Bateman, R. J., *et al*. Data-driven models of dominantly-inherited Alzheimer's disease progression. *Brain* 2018; 141(5), 1529–1544. <https://doi.org/10.1093/brain/awy050>
4. Firth, N. C., Primativo, S., Brotherhood, E., Young, A. L., Yong, K., Crutch, S. J., *et al*. Sequences of cognitive decline in typical Alzheimer's disease and posterior cortical atrophy estimated using a novel event-based model of disease progression. *Alzheimers dement* 2020, 16(7), 965–973. <https://doi.org/10.1002/alz.12083>
5. Zhang, W., Zhang, Z., Chao, HC. *et al*. Kernel mixture model for probability density estimation in Bayesian classifiers. *Data Min Knowl Disc* 2018; 32, 675–707. <https://doi.org/10.1007/s10618-018-0550-5>
6. Scott, D. W. (1979). On optimal and data-based histograms. *Biometrika* 1979, 66(3), 605-610.
7. Li, X., Wang, X., & Xiao, G. A comparative study of rank aggregation methods for partial and top ranked lists in genomic applications. *Briefings in bioinformatics* 2019, 20(1), 178–189. <https://doi.org/10.1093/bib/bbx101>
8. Lin, S.. Rank aggregation methods. Wiley Interdisciplinary Reviews: Computational Statistics 2010, 2(5), 555-570.
